# Radiation exposure induces cross-species temporal metabolic changes that are mitigated in mice by amifostine

**DOI:** 10.1038/s41598-021-93401-7

**Published:** 2021-07-07

**Authors:** Alexandra Crook, Aline De Lima Leite, Thomas Payne, Fatema Bhinderwala, Jade Woods, Vijay K. Singh, Robert Powers

**Affiliations:** 1 Department of Chemistry, University of Nebraska-Lincoln, Lincoln, NE 68588-0304 USA; 2 Nebraska Center for Integrated Biomolecular Communication, University of Nebraska-Lincoln, Lincoln, NE 68588-0304 USA; 3 Department of Pharmacology and Molecular Therapeutics, F. Edward Hébert School of Medicine, USUHS, 4301 Jones Bridge Road, Bethesda, MD 20814 USA; 4 Armed Forces Radiobiology Research Institute, USUHS, Bethesda, MD 20814 USA

**Keywords:** Biological techniques, Drug discovery, Environmental sciences, Natural hazards, Biomarkers, Diseases

## Abstract

Exposure to acute, damaging radiation may occur through a variety of events from cancer therapy and industrial accidents to terrorist attacks and military actions. Our understanding of how to protect individuals and mitigate the effects of radiation injury or Acute Radiation Syndrome (ARS) is still limited. There are only a few Food and Drug Administration-approved therapies for ARS; whereas, amifostine is limited to treating low dose (0.7–6 Gy) radiation poisoning arising from cancer radiotherapy. An early intervention is critical to treat ARS, which necessitates identifying diagnostic biomarkers to quickly characterize radiation exposure. Towards this end, a multiplatform metabolomics study was performed to comprehensively characterize the temporal changes in metabolite levels from mice and non-human primate serum samples following γ-irradiation. The metabolomic signature of amifostine was also evaluated in mice as a model for radioprotection. The NMR and mass spectrometry metabolomics analysis identified 23 dysregulated pathways resulting from the radiation exposure. These metabolomic alterations exhibited distinct trajectories within glucose metabolism, phospholipid biosynthesis, and nucleotide metabolism. A return to baseline levels with amifostine treatment occurred for these pathways within a week of radiation exposure. Together, our data suggests a unique physiological change that is independent of radiation dose or species. Furthermore, a metabolic signature of radioprotection was observed through the use of amifostine prophylaxis of ARS.

## Introduction

Global political unrest has highlighted the importance of understanding the short- and long-term effects of γ-radiation exposure to human health and survivability^1^. Radiation injuries fall into three categories: acute radiation syndrome (ARS), delayed or late arising pathologies, and chronic illnesses^2^. ARS arises from whole or partial-body exposure to a high dose of penetrating radiation over a short time period. The threats of accidental exposure, terrorist attack, or nuclear warfare is an ever-present danger, especially in the current political climate^3–6^. Efforts to understand the short- and long-term effects of radiation exposure to humans is an important national security interest. Thus, a rapid diagnostic tool and an effective treatment strategy for ARS is a paramount necessity^7^.

ARS biomarkers are an important tool for determining the effects of dose dependent radiation injury and may give valuable insights into disease progression and treatment options. Metabolomic biomarkers within accessible biofluids, such as blood and urine, offer a unique perspective of the downstream ARS effects^8–11^. Previous reports of mice exposed to ionizing radiation have shown dysregulation in serum metabolites including lipids and branched-chain amino acids as early as 24 h after exposure^12^. Analyses of mouse and non-human primate (NHP) biofluids have also shown a dysregulation in tricarboxylic acid (TCA) cycle metabolites and tryptophan metabolism^11,13–15^, a decrease in fatty acid oxidation and an increase in ketogenesis^15^, and a decrease in circulating blood-citrulline levels^10,16,17^. Biofluid metabolites associated with gut health and gastrointestinal (GI) inflammation have also been investigated for radiation induced dysregulation^11,18,19^. Similar investigations into mouse tissues have shown decreases in lipid and protein synthesis after radiation exposure^20,21^, or a reduced nucleotide metabolism from a low, long term dosage^22^. Likewise, metabolomics analysis of ionizing radiation in a clinical setting have shown a dysregulation in lipids and acyl-carnitines within 24 h of radiation exposure^23,24^. These prior studies have demonstrated the potential of metabolic dysregulation as a diagnostic marker of ARS.

Metabolic biomarkers also have the potential to provide a mechanism to evaluate radioprotective drugs for the prevention and mitigation of ARS^8,25,26^. Only four radiomitigators have been approved by the U.S. Food and Drug Administration (FDA) for use against hematopoietie-ARS^27^. No radioprotective agents have been approved for treating a patient prior to acute radiation exposure^27^. Amifostine, 2-(3-aminpropyl) aminoethylphosphorothioate, has been shown to reduce the effects of acute radiation exposure mainly through free radical scavenging and DNA protection^28–30^. Amifostine has only been FDA approved for limited, supervised clinical care^31–33^. The therapeutic status of amifostine presents a unique research opportunity—the evaluation of a known drug with radiomitigative properties as a potential radioprotective agent^34–37^. Thus, radiation induced metabolic changes may be useful for monitoring drug-efficacy and differentiating between radioprotective agents and radiomitigators.

Liquid chromatography-mass spectrometry (LC–MS)^34^ and nuclear magnetic resonance spectroscopy (NMR)^35,36^ based metabolomics have been extensively used to evaluate the physiological alterations that occur in a disease state. NMR or LC–MS are used separately to characterize the entire extracted metabolome from a biological or clinical sample. MS and NMR are inherently complementary methods for metabolomics and each technique detects a distinct set of metabolites, NMR observes the most abundant metabolites while MS detects readily ionizable metabolites as low as femtomolar concentrations^37–39^. Herein, we showcase an optimized extraction method for minimal blood samples that enabled a combined NMR and LC–MS metabolomics workflow. We report the time-dependency of radiation induced metabolic perturbations across two species and characterize the potential of amifostine as a radioprotective agent. We also identified potential metabolic biomarkers for ARS that may assist in evaluating drug efficacy.

## Results

### ARS animal models enabled search for radiation-induced biomarkers

**Figure 1:**
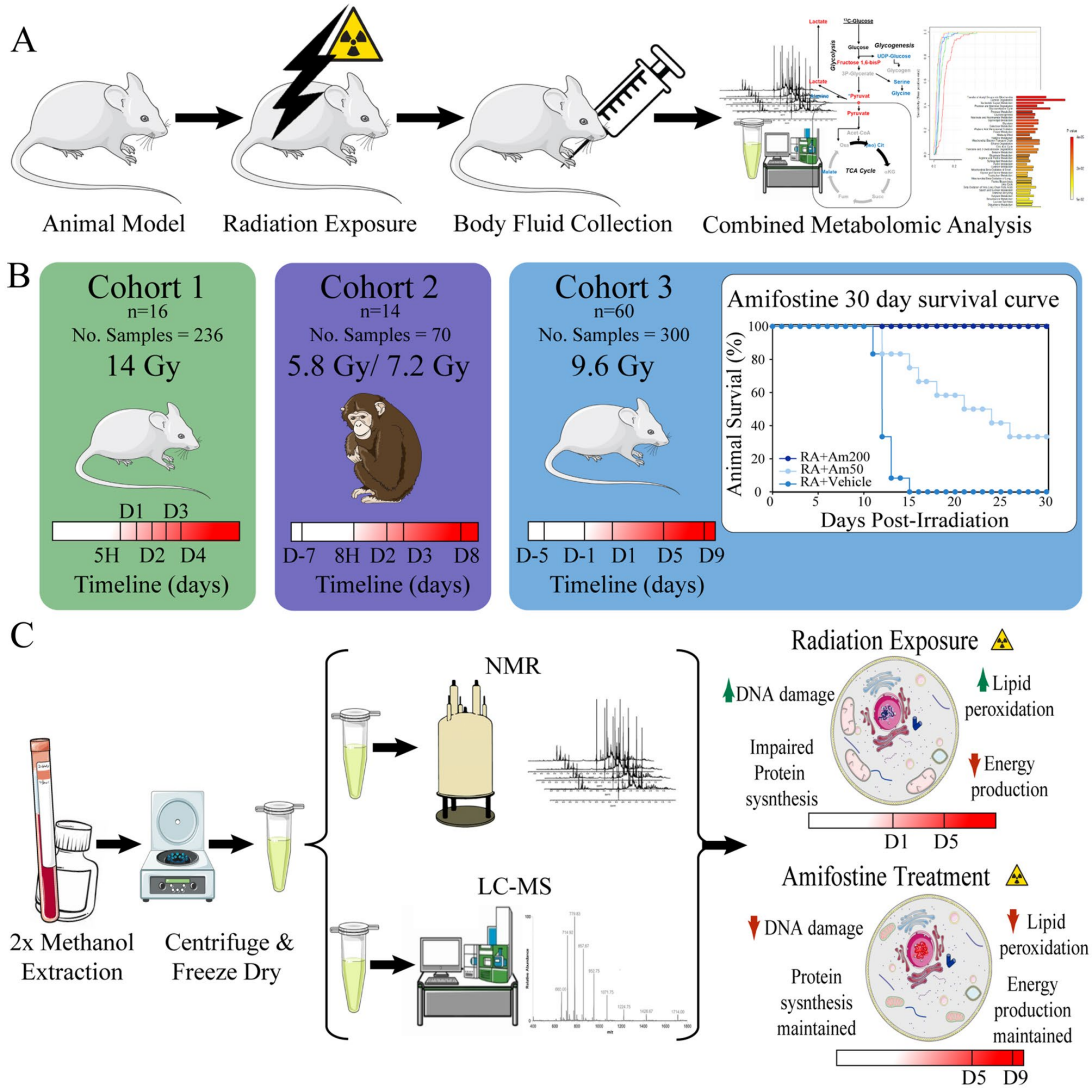
Combined metabolomics experimental design. (**A**) Study goals were to identify radiation biomarkers and monitor amifostine radioprotection. (**B**) Cohort variables included animal number(n), sample number (No. samples), radiation exposure, species, and biofluid collection time points. (**C**) A schematic of the combined NMR and LC–MS metabolomics approach. This figure was generated using medical images from Servier Medical Art (https://smart.servier.com/) under the Creative Commons License Attribution 3.0 Unported (CC BY 3.0).

The goal of this study was three-fold: (i) identify metabolite biomarkers of radiation exposure across multiple species, (ii) characterize temporal metabolic changes from acute radiation exposure, and (iii) evaluate amifostine as a radioprotector for ARS. As outlined in Fig. 1A, our experimental design consisted of biofluids collected from two ARS animal models pre- and post-radiation exposure. The species information, radiation exposure, and biofluid collection time points are summarized in Fig. 1B and Table 1. The metabolomes were extracted from the biofluids for a combined NMR and LC–MS analysis as shown in Fig. 1C.

**Table 1:** Demographics and sample information for the three ARS animal model cohorts**.**

Animal model	Fluid type	Radiation exposure	Time points				
Mouse n = 16 No. Samples - 236	Serum		5 h	Day 1	Day 2	Day 3	Day 4
		Control (CD)	24	22	24	24	24
		14 Gy	24	24	24	23	19
Non-human primates n = 14 No. Samples = 70	Serum		Day -7	8 h	Day 2	Day 3	Day 8
		5.8 Gy	7	7	7	7	7
		7.2 Gy	7	7	7	7	7
Mouse n = 60 No. Samples = 300	Blood		Day-5	Day-1	Day 1	Day 5	Day 9
		Am50	12	12	12	12	12
		Am200	12	12	12	12	12
		RAD (9.6 Gy)	12	12	12	12	12
		RAD+50 (9.6 Gy)	12	12	12	12	12
		RAD+200 (9.6 Gy)	12	12	12	12	12

Cohort 1 provided the basis to obtain a metabolomics signature of radiation exposure in mice.

Cohort 2 was utilized to determine the cross-species acute radiation induced metabolic response. Cohort 3 was used to determine the repeatability of the mouse metabolic changes resulting from irradiation, and, by inference, the consistency with the NHP dysregulated metabolism. It also extended the investigation into a correlation between radiation dosage and metabolome changes. Cohort 3 was also used to evaluate the dose-dependent radioprotective impact of amifostine treatment.

Thirty-day survival data on amifostine treatment of cohort 3 (Fig. 1B) demonstrates that at 9.6 Gy ^60^Co γ-radiation without preventive treatment resulted in 0% animal survival^21^. This same study showed that 50 mg/kg of amifostine treatment resulted in 40% survival at 30 days post-irradiation. Treatment with 200 mg/kg amifostine 30 min prior to radiation exposure showed the most promising results with 100% survival 30 days post-irradiation. This data demonstrates that amifostine treatment is an effective radioprotective agent.

Our metabolomic analysis further emphasized the temporal and dose dependent nature of radiation exposure and amifostine treatment (Fig. 1C). After radiation exposure, the three cross-species cohorts displayed dysregulated pathways between D1 and D5. These pathways included lipid degradation, impaired protein synthesis, and decreased TCA activity with downstream effects of amino acid metabolism and energy production. Pretreatment with amifostine (200 mg/kg) resulted in recovery of these dysregulated pathways between five and nine days after radiation exposure.

### Radiation exposure highlights temporal metabolic trajectories in mice

#### Cohort 1: mouse serum (14 Gy)

**Figure 2:**
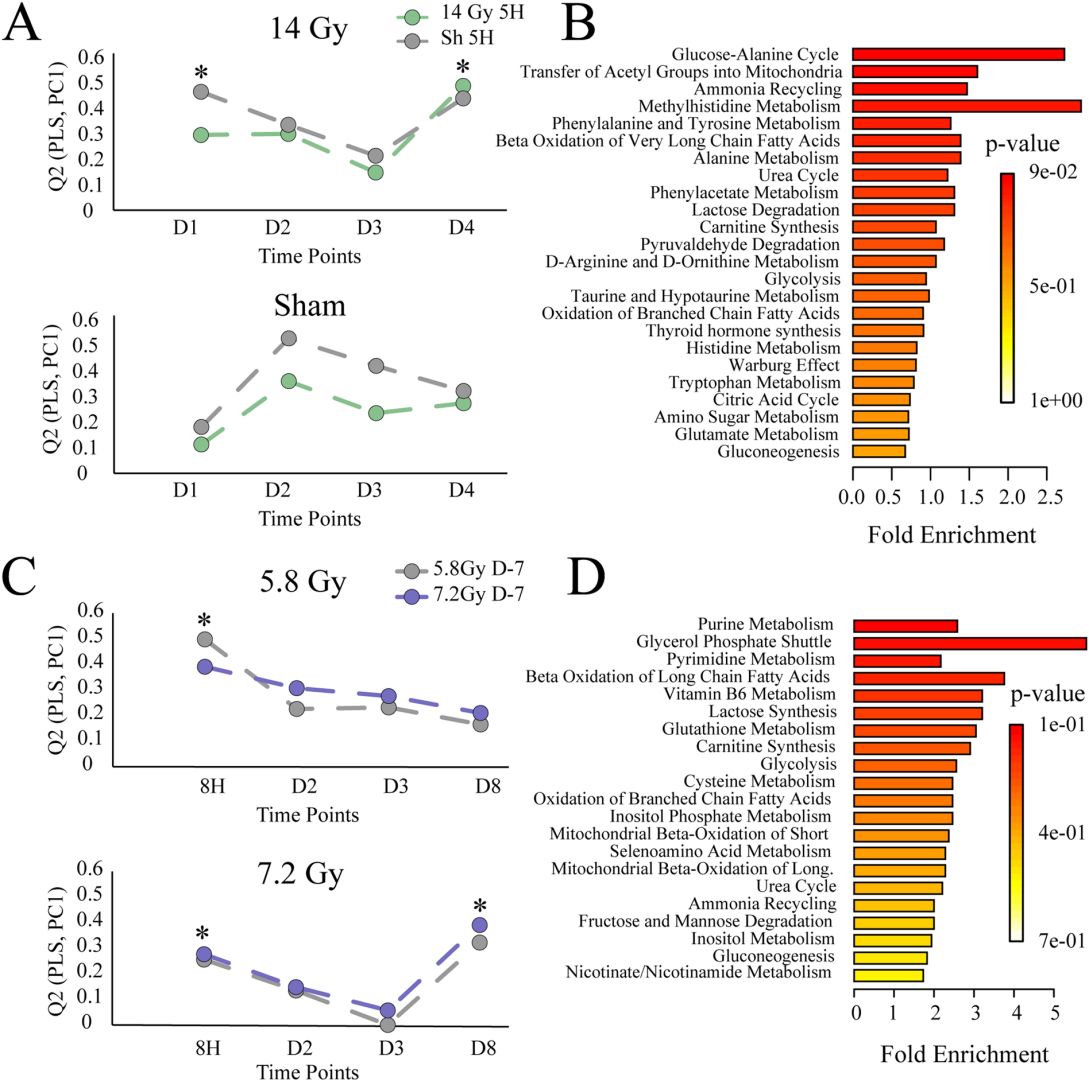
Combined metabolomic analysis following ^60^Co γ-radiation exposure. (**A**) Cohort 1 (mice) NMR metabolic trajectories of radiation (top) and Sham (bottom) calculated by PLS from baseline (5H) and visualized across time (*Q*^2^). *Indicates model p-values < 0.05. (**B**) Cohort 1 (mice) MetaboAnalyst pathway enrichment with pathways displayed by fold enrichment. (**C**) Cohort 2 (NHP) metabolic trajectories show 5.8 Gy radiation (top) and 7.2 Gy radiation (bottom) calculated by PLS from baseline (5H) and visualized across time (*Q*^*2*^). *Indicates model p-values < 0.05 (**D**) Cohort 2 (NHP) MetaboAnalyst pathway enrichment analysis with pathways displayed by fold enrichment.

For both NMR and LC–MS, all radiation (Gy) and Sham (Sh) time-points [day 1 (D1), day 2 (D2), day 3 (D3), day 4 (D4)] were modeled separately against baseline norm of 5 hours (5H) with projection to latent structures (PLS). The resulting *Q*^2^ statistics were then visualized across time (Fig. 2A, Supplementary Fig. S1A). The metabolic trajectory was nicely reproduced for Gy when the baseline norm of Gy5H was replaced with Sh5H.

Preliminary analyses of NMR data from mice serum samples showed that 14 Gy exposed and Sham mice experienced different temporal trajectories. This is evident by comparing the top (14 Gy) and bottom (Sh) panels in Fig. 2A. Exposure to 14 Gy radiation invoked a dual response in the NMR mouse metabolome, which occurred at day 1 (D1, p = 2.83 × 10^–8^) and day 4 (D4, p = 1.33 × 10^–10^) post-irradiation. Sham intervention exhibited only a single response at D2 (*Q*^2^ > 0.40). A similar response was observed in the analysis of LC–MS data (Supplementary Fig. S1A).

Shared and Unique Structures (SUS) principles was utilized to further characterize the significant responses between 5H baselines and the Gy or Sham groups, as well as comparisons between GyD4, GyD1 or ShD2 (Supplementary Fig. S1B). The SUS-plots show shared structures along the diagonal and unique structures off-diagonal. As expected, each within comparison displayed abundant shared structures, and good correlation (pcorr), particularly for a VIP > 1. Comparisons between GyD1 and GyD4 displayed abundant unique structures, while the comparisons between GyD1 and ShD2 displayed abundant shared structures along the diagonal. The latter comparisons inferred the Sham D2 response was independent of radiation and was likely due to a delayed stress response. LC–MS data highlighted similar effects of interest at GyD1 (p = 1.22 × 10^–10^) and GyD4 (p = 1.11 × 10^–16^). A similar SUS pattern could also be observed, with a stronger correlation between GyD1 and ShD2 than between GyD1 and GyD4 (Supplementary Fig. S1B). Thus, subsequent analyses were constrained to GyD4 vs 5H as the main radiation response.

A total of 34 NMR and 1630 MS features were found to be significant to group separation and radiation perturbation (VIP > 1) in significant PLS models (*Q*^2^ > 0.40, p < 0.05). Univariate statistics were adopted to further filter significant NMR and LC-MS spectral features and calculate traditional T-test statistics (p < 0.05) and fold changes (FC > 1) for GyD4 as shown in Supplementary Fig. S1C). A total of 29 NMR and 1584 MS spectral features retained differential significance from the baseline (5H) to GyD4. Metabolite identification resulted in a total of 95 putative metabolite identifications (12 from NMR and 83 from MS) as shown in Supplementary Table S1. Heatmaps of NMR and MS features show distinct clustering of metabolites at the 5H and D4 time points (Supplementary Fig. S1D). A MetaboAnalyst^40^ pathway enrichment analysis of these 95 metabolites resulted in 49 potentially dysregulated metabolic pathways as shown in Fig. 2B and Supplementary Table S2.

### Metabolic trajectories in NHP are dose dependent

#### Cohort 2: NHP serum (5.8 Gy and 7.2 Gy)

Both LC–MS and NMR, and all 5.8 Gy and 7.2 Gy time points [8 hours (8H), day 2 (D2), day 3 (D3), day 8 (D8)] were modeled with PLS against the baseline norm of 7 days prior to irradiation (D-7). The resulting *Q*^2^ statistics from the LC–MS or NMR data sets were then visualized across time to map trajectories (Fig. 2C, Supplementary Fig. S2A). The metabolic trajectories were reproduced for the 5.8 Gy and 7.2 Gy conditions when the D-7 baseline norms were interchanged.

As shown in Supplementary Fig. S2A, the NMR trajectories showed that NHP experienced a single response to both radiation exposures (5.8 Gy and 7.2 Gy). The extent of the perturbation for 5.8 Gy was delayed to D2 (p = 2.25 × 10^–3^), compared to 8H (p = 1.62 × 10^–2^) for 7.2 Gy. This delayed response suggests a dose-dependent metabolic response to radiation exposure. A supplementary pairwise time-point comparison was made between the 5.8 Gy and 7.2 Gy data sets. The orthogonal projection to latent structures-discriminant analysis (OPLS-DA)  models were only valid for the D-7 and 8H time points. At all other time-points, 5.8 Gy and 7.2 Gy irradiated NHP could not be discriminated. SUS principles were applied to further characterize the significant responses for the D-7 within comparisons (5.8 Gy and 7.2 Gy), and the comparisons between 5.8 Gy D2 and 7.2 Gy 8H (Supplementary Fig. S2B). Each D-7 within comparison displayed an abundant shared structure. The comparison between 5.8 Gy D2 and 7.2 Gy 8H displayed only minimal shared structures, which is indicative of a response independent of radiation exposure and likely due to stress (Supplementary Fig. S2B). A response to radiation would be expected to possess ample consistency between the two doses (5.8 Gy and 7.2 Gy). With the shared structures absent between comparisons, NMR alone appears insufficient to characterize the metabolic changes in NHP serum due to radiation exposure at 5.8 Gy and 7.2 Gy. Conversely, LC–MS data showed that 5.8 Gy and 7.2 Gy irradiated NHP experienced different temporal trajectories (Fig. 2C). Exposure to 7.2 Gy radiation invoked a dual response at 8H (p = 4.70 × 10^–3^) and D8 (p = 5.30 × 10^–3^). Conversely, 5.8 Gy only exhibited a single response at 8H (*Q*^2^ > 0.30). This observation is consistent with a metabolic dose dependent response.

A subsequent pairwise time-point comparison between the 5.8 Gy and 7.2 Gy data sets was conducted using OPLS-DA. The resulting OPLS-DA models proved valid for D-7, 8H and D8. At all other time-points, 5.8 Gy and 7.2 Gy exposed NHP could not be discriminated. When filtered for VIP > 1, SUS correlations were stronger between 5.8 Gy 8H and 7.2 Gy 8H than between 7.2 Gy 8H and 7.2 Gy D8 (Supplementary Fig. S2C). Thus, subsequent metabolomic analysis was constrained to a comparison between D-7 and 7.2 Gy D8 as the main response to radiation exposure.

Group separation in a valid PLS model resulted in 406 significant MS features, where 92 MS features retained differential significance from the baseline (D-7) to 7.2 Gy D8 after T-test and fold change filtering (Supplementary Fig. S2E). The 16 putative metabolites identified from these MS features are shown in Supplementary Table S3. A heatmap of MS features show distinct clustering of metabolites at the 7.2 Gy D-7 and D8 time points (Supplementary Fig. S2E). MetaboAnalyst pathway enrichment analysis identified 29 potentially dysregulated metabolic pathways (Fig. 2D, Supplementary Table S4).

### Amifostine imparts radioprotection in a dose dependent manner

#### Cohort 3: mouse blood (9.6 Gy)

The strategy for data analysis remained consistent with the previous two cohorts. Both NMR and LC–MS, and all time-points [day-1 (D-1), day 1 (D1), day 5 (D5), day 9 (D9)] were modeled with PLS against the baseline norm of day-5 (D-5). The resulting *Q*^2^ statistics were then visualized across time to map trajectories (Supplementary Fig. S3A,B). Once again, the metabolic trajectories could be reproduced for both conditions when the baseline norms were interchanged. The NMR results showed that an exposure to 9.6 Gy (0 mg/kg amifostine) invoked a single, incremental response throughout time that peaked at D9 (p = 1.74 × 10^–14^). LC–MS data confirmed an equivalent metabolic time trajectory towards radiation response at D9 (p = 6.39 × 10^–4^) as shown in Supplementary Fig. S3A.

The sharpest inclines (metabolome changes) occurred at post-irradiation time points between D1 vs D5 and D5 vs D9. Interestingly, SUS analysis of the NMR and LC–MS data sets displayed abundant shared structure, though anti-correlated (pcorr), between the significant responses, particularly with a threshold set at VIP > 1 (Supplementary Fig. S3C). Thus, subsequent analysis was constrained to a comparison of 9.6 Gy D1 to D5 as the main response to radiation.

Group separation in a valid PLS model resulted in 77 NMR and 2969 MS significant features, where 65 NMR and 916 MS features retained differential significance from D1 to D5 after T-test and fold change filtering (Supplementary Fig. S3D). The 34 putative metabolites identified from these features as shown in Supplementary Table S5. A heatmap of radiation exposure showed metabolite clustering among both LC–MS and NMR at time points RAD D1 vs D5 (Supplementary Fig. S4A). MetaboAnalyst pathway enrichment resulted in 88 potentially dysregulated metabolic pathways (Supplementary Fig. S4B, Supplementary Table S6).

Following radiation exposure (9.6 Gy), profiling of whole blood by NMR and LC–MS showed that untreated (0 mg/kg) and amifostine treated (50 mg/kg and 200 mg/kg) mice experienced different, non-stationary metabolic time trajectories (Supplementary Fig. S3A,B). While trajectories at the low amifostine dose (50 mg/kg) could not be discriminated from untreated controls, the high amifostine dose (200 mg/kg) reversed progression back towards the baseline norm (D-5) by D9 (NMR: p = 1.60 × 10^–7^ LC–MS: p = 2.10 × 10^–2^) as shown in Figures S3B. These results are consistent with a metabolic dose-dependent response to amifostine treatment.

Group separation in a valid PLS model resulted in 64 NMR and 3568 significant MS features, where 43 NMR and 1986 MS features retained differential significance from D5 to D9 after T-test and fold change filtering (Supplementary Fig. S3D). The 52 putative metabolites identified from these features as shown in Supplementary Table S5. A heatmap analysis showed metabolite clustering among both LC–MS and NMR at RAD+200 D5 vs D9 (Supplementary Fig. S4C). MetaboAnalyst pathway enrichment resulted in 83 potentially dysregulated metabolic pathways (Supplementary Fig. S4D, Supplementary Table S8).

## Discussion

The threat of nuclear warfare and terrorism is an ever-present concern in the current political landscape, and as a result there is a paramount need for ARS diagnostic information and therapeutic treatments^1^. Despite continuous research efforts since World War II that demonstrated the destructive potential of radioactive fallout, there are no FDA-approved radioprotective drugs available to prevent ARS^27^. To address these issues, three distinct cohorts consisting of two species were evaluated for a consistent metabolic signature of radiation exposure (Fig. 1). A combined NMR and LC–MS metabolomics platform provided a comprehensive coverage of the mice and NHP metabolomes after exposure to a potentially lethal dose of radiation.

**Figure 3:**
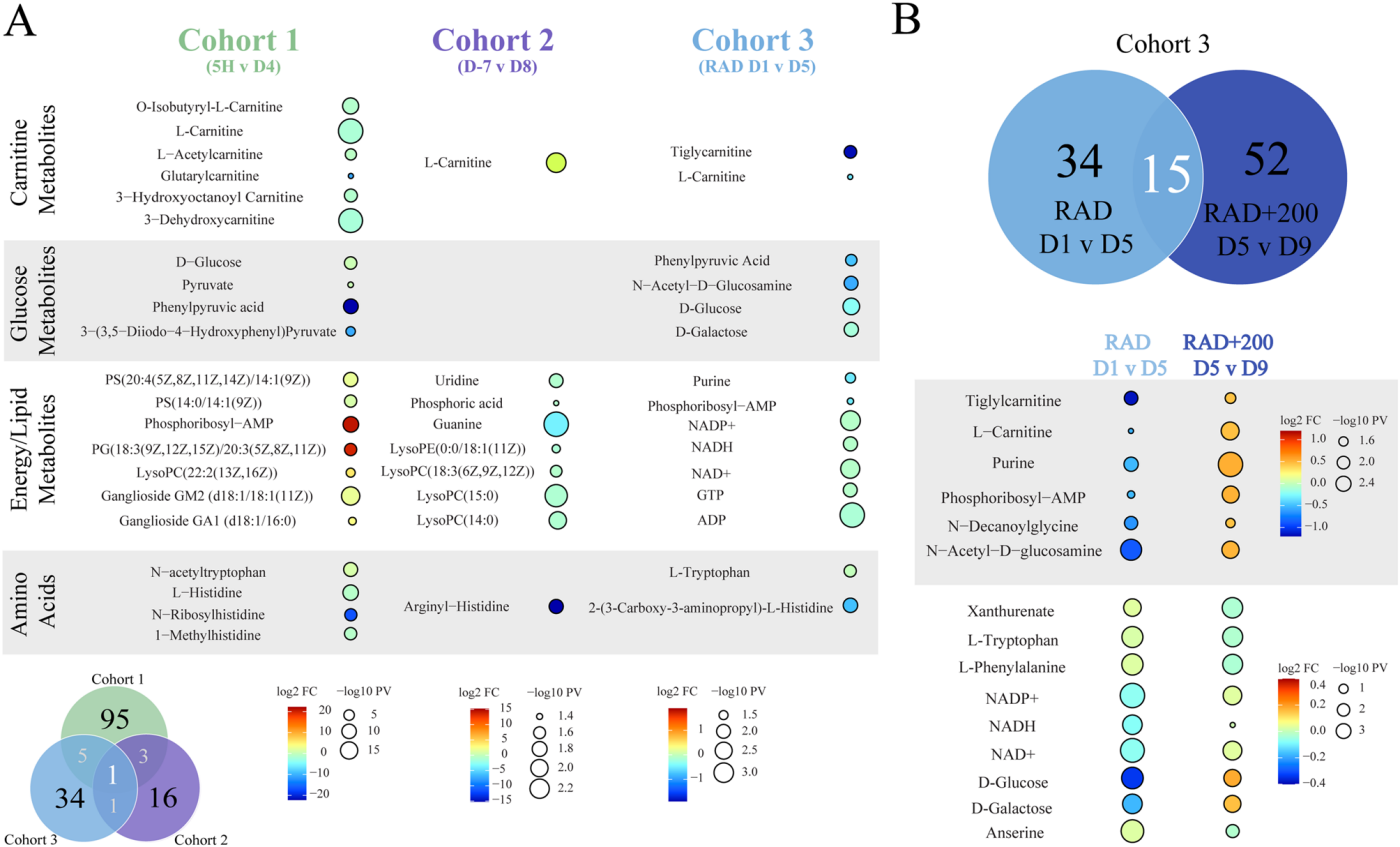
Overview of metabolite changes resulting from exposure to ^60^Co γ-radiation. (**A**) Venn diagram summarizing dysregulated metabolites from the three cohorts and raindrop plot illustrations of radiation effects in cohorts 1 to 3. (**B**) Venn diagram summarizing the metabolic pathways from cohort 3 that were dysregulated with or without amifostine treatment (200 mg/kg) and raindrop plot illustrations of shared metabolites between RAD D1vD5 and RAD+200 D5vD9. Raindrop plot illustration show model changes by fold change (logFC) and p-value (− log10 PV).

**Figure 4:**
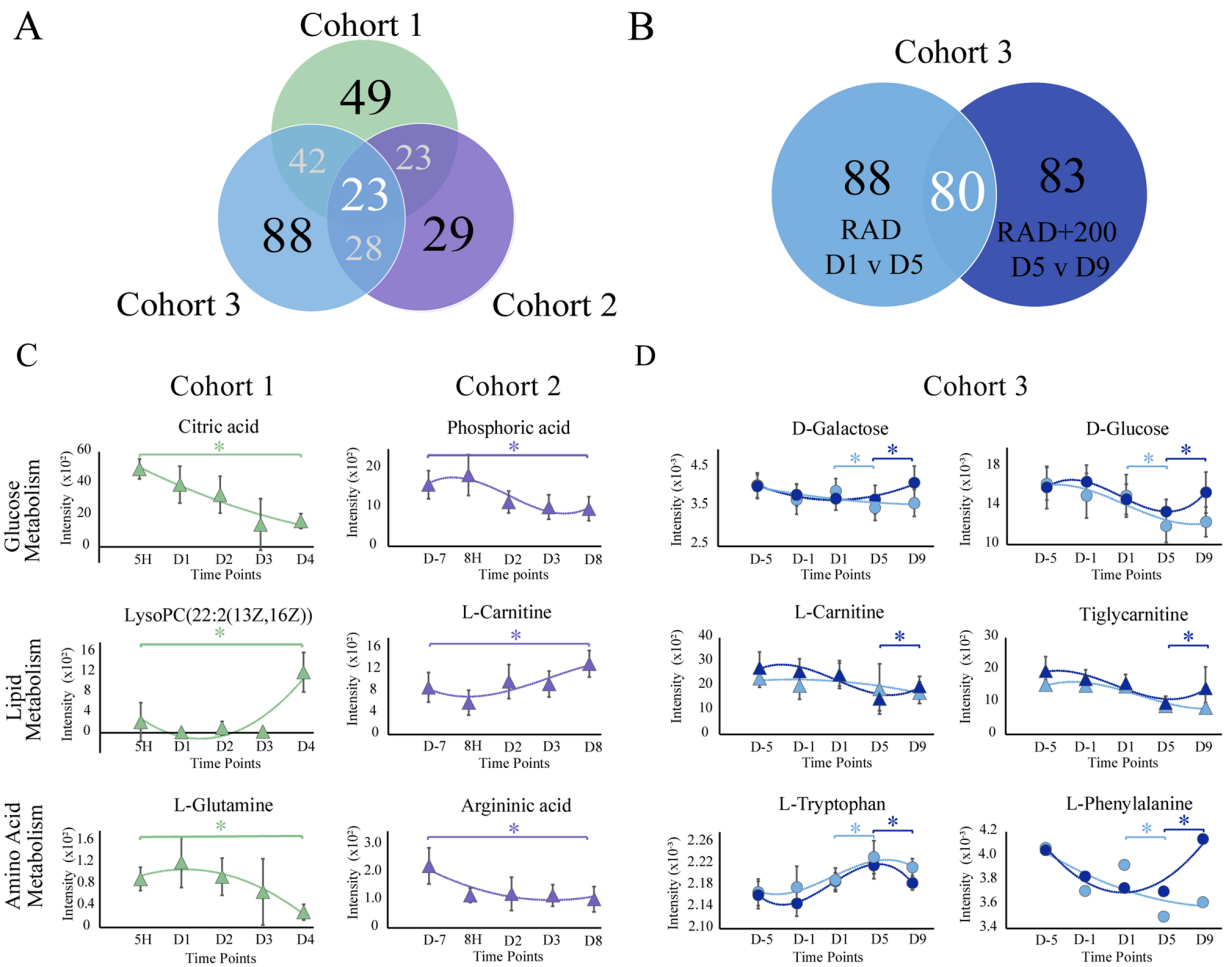
Overview of metabolic pathway changes resulting from exposure to ^60^Co γ-radiation. (**A**) Venn diagram summarizing dysregulated metabolic pathways from the three cohorts. (**B**) Venn diagram summarizing the metabolic pathways from cohort 3 (mice) that were dysregulated with or without amifostine treatment (200 mg/kg). (**C**) Representative metabolite trajectories from the LC–MS (triangles) and NMR (circles) data sets within cohort 1 or 2. *Denotes p-values < 0.05 and VIP > 1. (**D**) Representative metabolite trajectories from the LC–MS (triangles) and NMR (circles) data sets within cohort 3: radiation exposure (RAD D1 vs D5, gray) and radiation exposure with amifostine pretreatment (RAD+200 D5 vs D9, dark blue). *Denotes p-values < 0.05 and VIP > 1.

Overall, 95 metabolites were putatively identified in mice (cohort 1) with contributions from both NMR (12, 13%) and LC–MS (83, 87%) (14 Gy, Supplementary Table S3). The replicate ARS mouse model (cohort 3) also focused on detecting biomarkers of radiation exposure (9.6 Gy, Supplementary Table S5) as well as radiation mitigation from amifostine treatment (200 mg/kg, Supplementary Table S7). A total of 34 metabolites were dysregulated due to radiation exposure (RAD D1vD5) consisting of 22 metabolites identified by NMR (65%) and 12 metabolites identified by LC–MS (35%). A total of 52 metabolites were affected by amifostine treatment (RAD+200 D5vD9) with contributions from both NMR (14, 27%) and LC–MS (38, 73%). Environmental metabolic perturbations were apparent in the NHP (cohort 2) NMR data as demonstrated by the lack of a consistent metabolic signature observed in the SUS analysis (Supplementary Fig. S2B). Analysis was limited to LC–MS, which identified 16 metabolites as potential biomarkers of radiation (7.2 Gy, Supplementary Table S3). Notably, and as clearly illustrated by Figs. 2, 3, 4, these metabolic changes were temporally dependent in addition to being impacted by the species, radiation dose, or amifostine treatment.

There was a limited consistency in metabolite identities across the three cohorts. In total, cohorts 1 and 3 (mice) shared five metabolites, while cohort 1 (mice) and cohort 2 (NHP) shared three metabolites (Fig. 3A). Only one metabolite, l-carnitine, was consistently perturbed in all three cohorts. Cohort 1 (mice) contained six carnitine metabolites that were consistently down regulated at GyD4 compared to baseline Gy5H (Supplementary Table S1). Downregulation of carnitine metabolites was again observed in cohort 3 as shown in Fig. 3A (mice) RAD D1vD5, where two carnitine metabolites were observed (Supplementary Table S5). As shown in Figs. 3B and 4D, the carnitine metabolites from cohort 3 (mice) recovered toward baseline levels at D5vD9 in the RAD+200 amifostine treatment group (Fig. 3B). However, an increase in carnitine levels at D8 compared to baseline D-7 levels occurred for cohort 2 (NHP) (Figs. 3A and 4C). This increase in carnitine levels had been previously observed in NHP samples, which was attributed to renal failure and a resulting increase in cellular leakage^14,41^. Furthermore, a prolong increase in carnitine levels suggested deficiencies in fatty acid oxidation. All three cohorts exhibited a significant dysregulation in both carnitine metabolism and downstream lipid biosynthesis; however, the differences in carnitine metabolism between the cohorts may be attributed to species and dose variations. Notably, these issues may need further investigation. Despite these limited consistencies in identified radiation-induced metabolite perturbation, the three cohorts exhibited a consistency in dysregulated metabolic pathways. A total of 23 metabolic pathways were found to be uniformly perturbed in the three cohorts due to radiation exposure (Fig. 4A). The major dysregulated pathways based on fold change and p-value were glucose metabolism, de-novo lipid synthesis and metabolism, and amino acid metabolism (Supplementary Tables S2, S4, S6).

In cohort 1, the mice were irradiated with 14 Gy (LD_50/30_ for CD2F1 mice is roughly 8.6 ^60^Co γ-radiation) while in cohort 3 the mice received a lower irradiation dose of 9.6 Gy (LD_90/30_). Both mouse models showed significant radiation induced metabolic changes 4 to 5 days after exposure (Fig. 1A, Supplementary Figs. S1A, S3A,B). A comprehensive analysis indicated 42 pathways were similarly dysregulated between the two cohorts (Fig. 4A). Alterations to amino acid synthesis and glucose metabolism was a common metabolic response to radiation. Cohorts 1 and 3 (mice) showed a decrease in metabolites such as glucose, carnitine, and phenylpyruvic acid due to the radiation exposure (Fig. 3A and Supplementary Tables S1, S5). As illustrated by representative relative metabolite concentrations in Fig. 4C, radiation exposure induced a decrease in glucose metabolism and amino acid metabolism, and an increase in lipid synthesis throughout cohort 1 (mice), which received a high dose of radiation at 14 Gy. Conversely, cohort 3 (mice), which received a smaller dose of radiation at 9.6 Gy, demonstrated a decrease in glycolysis and energy metabolites (Fig. 3A) within the first 5 days after radiation exposure. This suggested the dysregulation in glucose metabolism was dependent on the radiation dose.

Similar to the decrease in energy metabolites observed in cohort 3 (mice) shown in Fig. 3A, cohort 2 (NHP) showed a significant decrease in lipid metabolites within 7 days after radiation exposure (Fig. 3A). Lipid metabolism involves the oxidation of fatty acids to generate energy, the decrease in both lipid and energy metabolites at lower doses of radiation suggested that de novo lipid biosynthesis and maintenance were significantly dysregulated within the first week of acute radiation exposure. Conversely, lipid metabolism increased in cohort 1 (mice), which were exposed to a higher dose of radiation (Fig. 3A).

Overall, our results are consistent with prior studies using either mouse or NHP ARS models^12–15,19,41–44^. For example, LC–MS and NMR metabolic studies of mouse ARS models have also observed perturbations in lipid synthesis and central carbon metabolism^12,13,42^. Khan et al*.* observed a decrease in the glucose signal within 3 days after 3, 5, and 8 Gy radiation doses^12^. An increase in lactate production was also observed. These radiation-induced metabolite changes were attributed to an overall increase in anaerobic metabolism. Kurland et al*.* on the other hand observed a significant increase in glycolysis metabolites within 24 hours after 50 Gy whole liver irradiation on mice^13^. Taken together, a radiation dose appears to have a significant effect on glucose metabolism. While low dose radiation appears to decrease glycolysis and increase anaerobic metabolism, a high dose of acute radiation increases glycolysis and lipid biosynthesis within the first week after radiation exposure.

Ghosh et al*.* analyzed mouse GI tissues with LC–MS s four days after irradiation and detected a significant dysregulation in amino acid metabolism, including glycine, serine, and threonine, as well as perturbations to pyruvate metabolism and the TCA cycle^42^. Similarly, Khan et al*.* utilized 1D ^1^H NMR to identify metabolic perturbations in mouse serum and liver tissue^12^. An increase in branched chain amino acids such as alanine and glutamine was observed five days after radiation exposure^12^. Ghosh et al*.* and Khan et al*.* also identified a significant dysregulation in mouse lipid metabolism in response to acute irradiation^42^. While Ghosh et al*.* reported a down regulation of phospholipids in a dose dependent manner, Khan et al*.* observed an increase in the levels of choline and phosphocholine^12,42^. These data, along with our analysis, implies that a radiation dose has a significant impact on glucose metabolism, phospholipid synthesis, and cell membrane regeneration.

Similar metabolic perturbations have been reported in NHP serum samples analyzed by LC-MS^14,15,41,43^. Pannkuk et al*.* observed a down regulation of amino acid metabolism, such as glutamate, alanine, and arginine, and in carnitine metabolism ten days after 10 Gy radiation exposure^41^. In a follow-up study, Pannkuk et al*.* identified a dysregulation in fatty acid β-oxidation, including increased levels of carnitine and propionylcarnitine 24 h after radiation exposure^14^. Furthermore, perturbations in amino acid metabolism, including a tryptophan dysregulation, were associated with a 6.5 Gy dose (LD_25-50/60_) of Co^60^ γ-radiation at 12 to 24 h post-irradiation exposure^14^. Similar to these results, our previous NHP analysis showed increased levels of carnitine (Fig. 3A). In addition, we observed a downregulation of phospholipids and purine metabolism, and evidence for DNA damage based on decreased levels of guanine uridine, hypoxanthine, and 2-hydroxyadenine as shown in Fig. 3A. We also observed tryptophan metabolism to be significantly perturbed across radiation doses and species (Fig. 3). Importantly, tryptophan metabolism was observed to return to normal levels following amifostine treatment.

The radioprotective potential of amifostine was also evaluated in cohort 3 (mice). Amifostine improved the 30-day survival rate of mice in a dose dependent manner (Fig. 1B)^27^. Mice pre-treated with 200 mg/kg survived at least 30-days, while untreated mice died within 15 days. While the metabolomic signature of amifostine at the lower dose (50 mg/kg) could not be distinguished from the control radiation samples, the amifostine treatment at the higher dose (200 mg/kg) showed a marked divergence from the control samples. This dose dependence was also observed by Cheema et al.^21,45^. As shown in Fig. 3B, analysis of RAD D1vD5 provided 34 significantly altered metabolites and RAD+200 D5vD9 provided 38 significantly altered metabolites. Furthermore, 15 metabolites were shared between the RAD D1vD5 and RAD+200 D5vD9 groups. As summarized in Fig. 3B, 14 of these 15 shared metabolites showed an altered fold change when comparing RAD D1vD5 to RAD+200 D5vD9. It is evident from the raindrop plot illustrations that these metabolites were downregulated at RAD D1vD5 while there was an increase in metabolite levels between RAD+200 D5vD9. These altered metabolites included d-glucose, l-carnitine, and NAD^+^, which are involved in glycolysis, energy metabolism, and amino acid metabolism. This analysis suggests that recovery of energy metabolism, lipid biosynthesis, carnitine metabolism, and glycolysis are significantly affected by acute irradiation. Furthermore, pre-treatment with a radioprotective agent can alter the course of these radiation-induced metabolites. A total of 80 metabolic pathways were similarly dysregulated between radiation exposure (RAD D1vD5) and 200 mg/kg amifostine treatment prior to radiation exposure (RAD+200 D5vD9) (Fig. 4B). While many of the pathways were shared between the RAD and RAD200 groups, the SUS analysis (Supplementary Fig. S3C) of the two groups and the shared metabolites (Fig. 3B) demonstrated a negative relationship as would be expected by a recovery treatment. Amifostine administration prior to radiation exposure resulted in a return to metabolic baseline at time points D5 to D9 (Supplementary Fig. S3A,B).

While the study terminated at D9, it is evident from these PLS time course data for both NMR and MS that the RAD *Q*^2^ results show an increased trajectory from D1 through D9 suggesting that the animal models were more dissimilar from the control at D-5 (Supplementary Fig. S3A). The RAD+200 trajectory changed between D5 and D9, and the *Q*^2^ results both decreased for the 200 mg/kg amifostine treatment, which suggests a closer similarity with control samples (Supplementary Fig. S3B). The metabolomic trajectory for RAD+200 shows a significant (p-value < 0.05) shift from D5 to D9 that suggests a return to levels observed at D-5 (Fig. 4D). Overall, our data demonstrates a time delayed metabolomics effect from radiation exposure in both mice and NHP. The most prevalent changes occur in glucose metabolism and downstream amino acid synthesis, as well as lipid synthesis and carnitine metabolism.

## Conclusions

Overall, a consistent and reproducible metabolomic signature of radiation exposure was detected across two species, multiple radiation doses, and multiple time-points. Metabolic pathways were rapidly perturbed in a dose dependent manner within a few days of radiation exposure. Twenty-three metabolomic pathways were shown to be dysregulated by exposure to lethal doses of radiation, including glucose metabolism, amino acid metabolism and lipid synthesis. Furthermore, it is evident that radiation dose plays a significant role in the regulation of glycolysis, phospholipid and lipid biosynthesis, and purine metabolism within the first week after acute radiation exposure. Notably, amifostine pretreatment reversed ARS progression, and the temporal trajectories returned toward baseline levels. Thus, amifostine has a potential utility as a radioprotector. In total, a potential diagnosis of ARS may lay within the observed metabolomic response to radiation exposure, which may serve to monitor a patient’s response to ARS therapy.

## Materials and methods

### Animal models

Animal studies were conducted in a facility accredited by the Association for Assessment and Accreditation of Laboratory Animal Care (AAALAC)-International. All procedures involving animals were approved by the Institutional Animal Care and Use Committee (IACUC) and the Department of Defense Animal Care and Use Review Office (ACURO) for the NHP study. All studies were carried out in accordance with the recommendations in the *Guide for the Care and Use of Laboratory Animals* and in compliance with ARRIVE (Animal Research: Reporting of In Vivo Experiments) guidelines^46^.

The metabolomic study utilized three distinct animal cohorts (Table 1). Cohort one is comprised of male CD2F1 mice (*Mus musculus*) aged 12 to 14 weeks. A total of 236 serum samples were collected as previously described^47^. Sham (Sh, n = 8) mice were not exposed to radiation and test mice (Gy, n = 8) were exposed to a single dose of 14 Gy ^60^Co γ-radiation. The estimated LD_50/30_ for CD2F1 mice is 8.6 Gy ^60^Co γ-radiation. Serum samples were collected at five time-points: 5 Hours (5H), day 1 (D1), day 2 (D2), day 3 (D3) and day 4 (D4) post-radiation exposure. Each group contained eight biological samples with three analytical replicates for a total of twenty-four replicates per group with the following exceptions: Sh1 n = 22, GyD3 n = 23, and GyD4 n = 19 (Table 1).

Cohort 2 is comprised of male and female naïve rhesus macaques (*Macaca mulatta*, Chinese substrain) aged 3 to 5 years and weighing 3.6 to 8.4 kg. A total of 70 serum samples from 14 NHPs were collected as previously described^48^. NHPs (n = 7) were exposed to 5.8 Gy (LD_30/60_) or 7.2 Gy (LD_70/60_) ^60^Co γ-radiation at a rate of 0.6 Gy/min. Serum samples were collected at five time points: 7 days (D-7) prior to radiation exposure and four time-points post-radiation exposure at 8 h (8H), day 2 (D2), day 3 (D3), and day 8 (D8) (Table 1).

Cohort 3 is comprised of male CD2F11 mice aged 12 to14 weeks exposed to a single dose of 9.6 Gy (LD_90/30_) ^60^Co γ-radiation at a rate of 0.6 Gy/min^21^. Mice received a subcutaneous injection of either 50 mg/kg or 200 mg/kg of amifostine 30 min (± 10 min) before exposure to radiation. Amifostine administration to irradiated or unirradiated animals was exactly the same. The radiation dose was reduced to 9.6 Gy (LD_90/30_) to extend the study (survivability) out to Day 9 (D9) and to allow for a comparison to the Day 8 (D8) time point from the NHP cohort. A total of 300 whole blood samples were collected from 60 mice (n = 12) as previously described^21^. The five groups correspond to: (1) 50 mg/kg amifostine without radiation exposure (Am50), (2) 200 mg/kg amifostine without radiation exposure (Am200), (3) 50 mg/kg amifostine with radiation exposure (RAD+50), (4) 200 mg/kg amifostine with radiation exposure (RAD+200), and (5) only radiation exposure (RAD). Blood samples were collected at five time-points: at time of amifostine dosage day-5 (D-5), day-1 (D-1), day 1 (D1), day 5 (D5) and day 9 (D9) (Table 1).

All standard protocols, including metabolomic sample preparation, data collection, processing, and analyses, are available in supplemental material.

### Statistical model analysis

Without any prior knowledge, a hypothesis where height of perturbation (radiation exposure—effect of interest) equates to maximum observed metabolic difference was constructed. This simple assumption grants integration across datasets and time-points to trace metabolite responses to radiation exposure as a function of time. The assumption required a test condition to be assigned to each cohort. The test conditions were assigned as: 14 Gy (cohort 1), 5.8 Gy and 7.2 Gy (cohort 2), and 9.6 Gy (cohort 3). The NMR and LC–MS data across all time-points were modelled against a control free from perturbation, which served as the baseline. One predictive component (PC) was then used from the PLS model to construct metabolite trajectories. The resultant *Q*^2^ values from the PLS models were used as a proxy for metabolic perturbations and mapped across the time points to allow comparable trajectories to be interrogated. Significant PLS models (*Q*^2^ > 0.40, p < 0.05) were recalculated (Monte Carlo simulations) to evaluate and summarize parameters (*Q*^2^, pcorr, VIP) with confidence (95% CI). VIP > 1 was used to filter significant features. Univariate statistics were adopted to further filter significant NMR and MS spectral features and calculate traditional T-test statistics (p < 0.05) and fold changes (FC > 1).

## Supplementary Information

Supplementary Information.

## Data Availability

Research data are stored in an institutional repository and can be shared upon request to the corresponding author.
