# Supplementary material for: Radiation exposure induces cross-species temporal metabolic changes that are mitigated in mice by amifostine
Source: Sci Rep. 2021 Jul 7;11:14004. doi: 10.1038/s41598-021-93401-7 (PMC8263605; doi:10.1038/s41598-021-93401-7)
Supplement: Supplementary file 1 — Supplementary Information. [file 41598_2021_93401_MOESM1_ESM.pdf]

Supplemental Information for

Radiation Exposure Induces Cross-Species Temporal Metabolic Changes that are Mitigated in Mice by Amifostine

Alexandra Crook,[1,†] Aline De Lima Leite,[2,†] Thomas Payne,[2,†] Fatema Bhinderwala,[1,2] Jade Woods,[1] Vijay K. Singh,[3,4]* Robert Powers[1,2,]*

[1]*Department of Chemistry, University of Nebraska-Lincoln, Lincoln NE 68588*
[2] *Nebraska Center for Integrated Biomolecular Communication, University of Nebraska-Lincoln, Lincoln NE 68588*
[3]*Department of Pharmacology and Molecular Therapeutics, F. Edward Hébert School of Medicine, USUHS, Bethesda, MD 20814, USA*
[4]*Armed Forces Radiobiology Research Institute, USUHS, Bethesda, MD 20814, USA*

[†]equal contribution

*To whom correspondence should be addressed:

Robert Powers
University of Nebraska-Lincoln
Department of Chemistry
722 Hamilton Hall
Lincoln, NE 68588-0304

Email: rpowers3@unl.edu
Phone: (402) 472-3039
Fax: (402) 472-9402

Vijay K. Singh
Department of Pharmacology and Molecular Therapeutics
F. Edward Hébert School of Medicine, USUHS
4301 Jones Bridge Road, Bethesda, MD 20814

Email: vijay.singh@usuhs.edu
Phone: 301-295-2347
Fax: 301-295-6503

Statistical Analyses:

Thomas Payne
Email: 447999157376
Phone: paynet@hotmail.co.uk

Contents

Materials and Methods

- Chemicals
- Preparation of blood and serum samples for metabolomics analysis
- NMR data collection and processing
- LC-MS data collection and processing
- NMR and MS data analyses

Figures

Figure S1. Univariate Analysis of Cohort 1.

Figure S2. Univariate Analysis of Cohort 2.

Figure S3. Univariate Analysis of Cohort 3.

Figure S4. Heatmap and Pathway Enrichment of Cohort 3.

Figure S5. NMR and MS Metabolomics Trajectories of Amifostine Treatment.

Tables

Table S1. Perturbed Metabolites of Cohort 1

Table S2. Perturbed Pathways of Cohort 1.

Table S3. Perturbed Metabolites of Cohort 2

Table S4. Perturbed Pathways of Cohort 2.

Table S5. Perturbed Metabolites in Cohort 3 RAD D1vD5

Table S6. Perturbed Pathways of Cohort 3 RAD D1vD5.

Table S7. Perturbed Metabolites of Cohort 3 RAD+200 D5vD9.

Table S8. Perturbed Pathways of Cohort 3 RAD+200 D5vD9.

Materials and Methods

Chemicals

All chemicals were obtained from Sigma Aldrich (Milwaukee, WI) unless otherwise denoted. 3-(trimethylsilyl) propionic-2,2,3,3-D_4 acid sodium salt (98% D, TMSP) was purchased from Cambridge Isotopes (Andover, MA). Potassium phosphate dibasic salt (anhydrous, 99.1% pure) and monobasic salt (crystal, 99.8% pure) were purchased from Fisher Scientific (Fair Lawn, NJ).

Preparation of blood and serum samples for metabolomics analysis

Mouse (n=236) and NHP (n=70) serum samples, and mouse blood samples (n=300) were simultaneously prepared for LC-MS and NMR analysis as shown in **Figure 1C**. The preparation of blood samples were optimized from a previously published protocol.(1) 2.0% by volume of NaN_3 was added to each serum or blood sample to avoid bacterial growth. The protein component was precipitated by adding 100 μL (2x) of methanol to 50 μL of serum or blood. Deoxygenated methanol was used for the blood preparation to avoid unnecessary oxidation of the red blood cells. An increase in metabolite levels extracted from blood samples was previously observed with deoxygenated methanol.(1) Methanol was purged of oxygen by bubbling in nitrogen gas for 30 minutes.

The 1:2 mixtures were then vortexed for 10 seconds. Serum samples were incubated at room temperature for 5 min. Blood samples were sonicated in a water bath for 10 minutes at 4 °C, followed by incubation at -20 °C for 20 min. The samples were then centrifuged at 15,000 x *g* for 20 min at 4 °C to pellet the proteins. The supernatant was collected, transferred to an Eppendorf tube, and centrifuged again at 15,000 x *g* for 5 min at 4 °C. The supernatant was then divided 20%

and 80% for LC-MS and NMR samples, respectively. Samples were snap-frozen in liquid nitrogen. Methanol was evaporated by speed vacuum centrifugation (SpeedVac R Plus, Savant) and water was removed by lyophilization using FreeZone™ (Labconco, Kansas City, MO).

NMR data collection and processing

Dried serum or blood samples were reconstituted using 50 µL of 50 mM phosphate buffer in 100% D_2O at pH 7.2 (uncorrected) with 50 µM TMSP as an NMR chemical shift reference and internal standard. The samples were centrifuged at 15,000 x *g* for 20 min at 4 °C to remove any particulates, and the supernatant was transferred to 1.7 mm NMR tube for data acquisition. All NMR experiments were collected at 298K with a Bruker AVANCE III HD 700 MHz spectrometer equipped with a 5 mm quadruple resonance QCI-P cryoprobe (^{1}H, ^{13}C, ^{15}N and ^{31}P) with z-axis gradients. A SampleJet automated sample changer with Bruker ICON-NMR software was used to collect all data.

A one-dimensional (1D) ^{1}H NMR spectrum was collected for each sample. 1D ^{1}H NMR spectra were collected with 32K data points, a spectrum width of 8417.5 Hz, 256 scans, and 4 dummy scans using an excitation sculpting pulse sequence to remove the solvent peak.(2) In addition, a natural abundance 2D ^{1}H-^{13}C HSQC spectrum was collected for representatives from each group. The 2D ^{1}H-^{13}C HSQC spectra were collected with 64 scans, 16 dummy scans, and a 2 s relaxation delay. The spectra were collected with 2K data points and a spectrum width of 11160 Hz in the direct dimension, and 1024 data points and a spectrum width of 29052 Hz in the indirect dimension. The 2D ^{1}H-^{13}C HSQC spectra were collected with 25% sparsity using our deterministic non-uniform sampling (NUS) schedule.(3)

The 2D ^{1}H-^{13}C HSQC NUS spectra were reconstructed with the MDD algorithm.(4) Both 1D and 2D NMR spectra were processed using NMRPipe(5) or with our MVAPACK(6) software toolkit (http://bionmr.unl.edu/mvapack.php). Each 1D ^{1}H NMR spectrum was Fourier transformed, phased, referenced to TMSP and aligned with icoshift(7) to obtain a data matrix. The 1D ^{1}H NMR data matrix was then binned using the intelligent adaptive binning algorithm,(8) UV scaling, and normalized using the Probabilistic Quotient (PQ) method. The 2D ^{1}H-^{13}C NMR data was Fourier transformed and phased. The 2D ^{1}H-^{13}C HSQC spectra were visualized and peak-picked using NMRviewJ (version 8.0). Discriminatory features were identified to specific metabolites using Chenomx Suite 8.0, Human Metabolome Database (HMDB),(9) SpinAssin,(10) and BMRB.(11) A ^{1}H and ^{13}C chemical shift error of 0.08 and 0.25 ppm, respectively, was used to match experimental chemical shifts to reference spectra.

LC-MS data collection and processing

For LC-MS analysis, dried metabolome extracts were reconstituted in 20 μL of 0.1% formic acid and centrifuged at 15,000 x *g* at 4 °C for 10 min to remove particulates. Quality control (QC) samples were prepared by pooling 5 μL from each experimental metabolomics sample. An LC-MS spectrum was acquired for five QC samples prior to analyzing the metabolome extracts. In addition, two QC samples were analyzed after every 12 injections of experimental metabolomics samples to monitor system stability.

The metabolome analyses were performed using an ACQUITY Ultra-Performance Liquid Chromatography (UPLC) system (Waters, Milford, MA, USA) coupled to a Waters Xevo G2-XS

Q-TOF mass spectrometer (Waters Co., Milford, MA, USA.) with an electrospray ionization (ESI) mode. An ACQUITY UPLC HSS T3 C18 (1.0 × 50 mm, 1.8μm, Waters Co., Milford, MA, USA) column was used. Column and autosampler temperature were set to 40° C and 8 °C, respectively. The flow rate was set to 100 μL/min. The mobile phase was composed of 0.1% formic acid in water (A) and 0.1% formic acid in acetonitrile (B). 2 μL of a metabolome extract or a QC sample were injected and separated with a linear gradient program from 1% to 95% B in 7.30 min, held at 95% for 1.5 min and re-equilibrate for 1.2 min.

The ESI source was set to positive mode, and the capillary and cone voltages were set to 3.15 kV and 40 V, respectively. The source temperature was set to 120 °C, the cone gas flow was set to 50 L/h, and the desolvation temperature and flow rate were set to 300 °C and 857 L/h, respectively. Data collection was in the data-independent acquisition mode (MSE). The quadrupole was set to transfer all ions and alternate collision energy from low (4 eV) to high (ramped from 15 to 45 eV) energies with a *m/z* scan range of 50 to 2000 Da. The time of flight mass analyzer was calibrated with an external mass calibrant (Leucine-Enkephalin $[M+H]^+$=556.2771) infused via the Lock-mass channel.

Following acquisition, raw LC-MS data were imported into Progenesis QI v.2.1 (Non-linear Dynamics, Newcastle, UK) for automatic alignment, detection and deconvolution. 2D ion intensity maps were generated and the retention times were aligned using a QC pooled spectrum as a reference. The peak detection parameters were as follows: the sensitivity method value was set to 3, no minimum peak width and no retention time limits were used. Adducts of the same compound were automatically grouped during deconvolution using a list ($[M+H]^+$, $[M+Na]^+$, $[M+K]$,

$[M + NH_4]^+$, $[M + H–H_2O]^+$, $[M + H–2H_2O]^+$, $[M + ACN+H]^+$, $[M + CH_3OH+H]^+$ and $[M + ACN+Na]^+$) of adducts pre-defined from a raw data import. Normalization was computed to the mean log ratio (MLR) algorithm in Progenesis. Features not present in at least 50% of one experimental group or not present in the QC spectrum with a coefficient of variation (CV) of ≤ 30% were removed. Batch or run order variance were removed with LOESS QC corrections.(12) Putative annotation of metabolites was performed by MetaScope with a theoretical fragment search against HMDB.(9) The putative metabolite assignment was performed according to parameters, including Score, Fragmentation score, and Isotope similarity by Progenesis QI. Only statistically significant features were submitted for identification.

NMR and MS data analyses

All unsupervised and supervised multivariate statistics were conducted using MVAPACK in OCTAVE (4.4.1).(6) Unless otherwise stated, unit variance (UV) scaling was applied to both X and Y inputs before NIPAL implementation for Partial Least Squares/Projection to Latent Structures (PLS) and Orthogonal Projection to Latent Structures (OPLS) with successive iterations halted based on the cross-validated (7-fold CV) and fraction of Y variation modelled (Q^2). CV-ANOVA, permutation testing (n = 1000), and Variable Importance in Projection scores (VIP > 1) were used for model validation and evaluation, respectively. Shared and Unique (SUS) principles using p(corr) were adopted to facilitate model interpretation. All univariate statistics were conducted using 'Limma' or 'Stats' in R (3.5.2).(13) Unless otherwise stated, log2 transformations were applied before concurrent linear fits by generalized least squares and computed moderated T-statistics, F-statistics and log-odds by empirical Bayes moderation of standard errors to a common value (Limma). Respective p-values were adjusted for multiple testing by Benjamini–

Hochberg (BH) and q-values calculated to the expected false discovery rate (FDR), with significance to < 0.05. MetaboAnalyst software was used to access 'Pathways Analysis' and 'Pathways Enrichment' of all significantly altered metabolites (14).

References

1. Nagana Gowda GA & Raftery D (2017) Whole Blood Metabolomics by 1H NMR Spectroscopy Provides a New Opportunity To Evaluate Coenzymes and Antioxidants. *Analytical Chemistry* 89(8):4620-4627.
2. Nguyen BD, Meng X, Donovan KJ, & Shaka AJ (2007) SOGGY: Solvent-optimized double gradient spectroscopy for water suppression. A comparison with some existing techniques. *Journal of Magnetic Resonance* 184(2):263-274.
3. Worley B & Powers R (2015) Deterministic multidimensional nonuniform gap sampling. *Journal of Magnetic Resonance* 261:19-26.
4. Orekhov VY & Jaravine VA (2011) Analysis of non-uniformly sampled spectra with multi-dimensional decomposition. *Progress in Nuclear Magnetic Resonance Spectroscopy* 59:271-292.
5. Delaglio F, *et al.* (1995) NMRPipe: A multidimensional spectral processing system based on UNIX pipes. *Journal of Biomolecular NMR* 6(3):277-293.
6. Worley B & Powers R (2014) MVAPACK: a complete data handling package for NMR metabolomics. *ACS Chem Biol* 9(5):1138-1144.
7. Savorani F, Tomasi G, & Engelsen SB (2010) icoshift: A versatile tool for the rapid alignment of 1D NMR spectra. *J.ournal of Magnetic Resonance* 202(2):190-202.
8. Worley B & Powers R (2015) Generalized adaptive intelligent binning of multiway data. *Chemometrics and Intelligent Laboratory Systems* 146:42-46.

9. Wishart DS, *et al.* (2013) HMDB 3.0--The Human Metabolome Database in 2013. *Nucleic Acids Research* 41(Database issue):D801-D807.

10. Yamada S, *et al.* (2019) InterSpin: Integrated Supportive Webtools for Low- and High-Field NMR Analyses Toward Molecular Complexity. *ACS Omega* 4(2):3361-3369.

11. Ulrich EL, *et al.* (2008) BioMagResBank. *Nucleic Acids Research* 36(suppl_1):D402-D408.

12. Gagnebin Y, *et al.* (2017) Metabolomic analysis of urine samples by UHPLC-QTOF-MS: Impact of normalization strategies. *Anal Chim Acta* 955:27-35.

13. Dessau RB & Pipper CB (2008) "R"--project for statistical computing. *Ugeskr Laeger* 170(5):328-330.

14. Chong J, Wishart DS, & Xia J (2019) Using MetaboAnalyst 4.0 for Comprehensive and Integrative Metabolomics Data Analysis. *Current Protocols in Bioinformatics* 68(1).

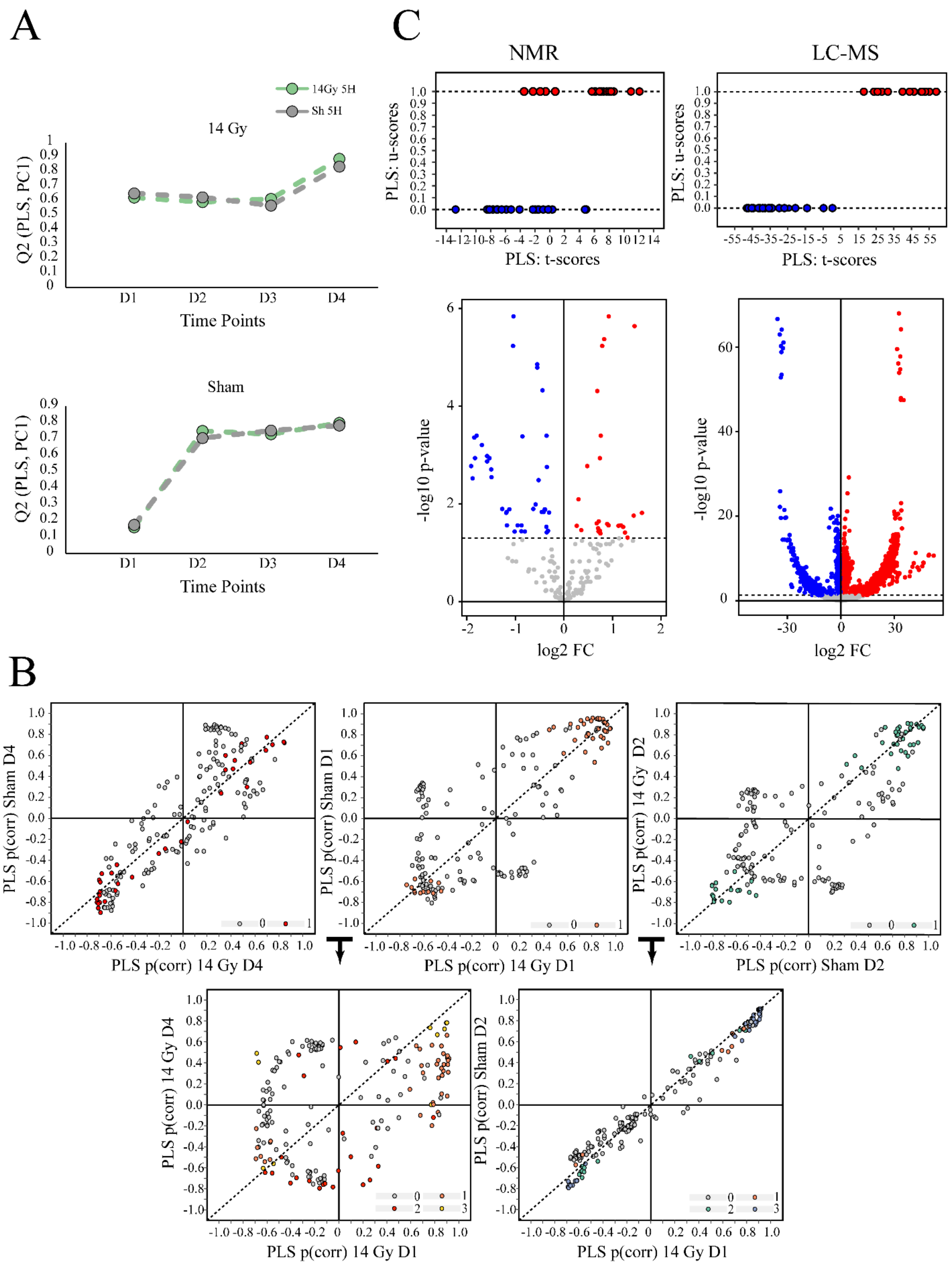
A
C
14Gy 5H
Sh 5H
14 Gy
Q2 (PLS, PC1)
D1
D2
D3
D4
Time Points
Sham
Q2 (PLS, PC1)
Time Points
NMR
LC-MS
PLS: u-scores
PLS: t-scores
-log10 p-value
log2 FC
B
PLS p(corr) Sham D4
PLS p(corr) 14 Gy D4
PLS p(corr) Sham D1
PLS p(corr) 14 Gy D1
PLS p(corr) 14 Gy D2
PLS p(corr) Sham D2
PLS p(corr) 14 Gy D4
PLS p(corr) 14 Gy D1
PLS p(corr) Sham D2
PLS p(corr) 14 Gy D1

D

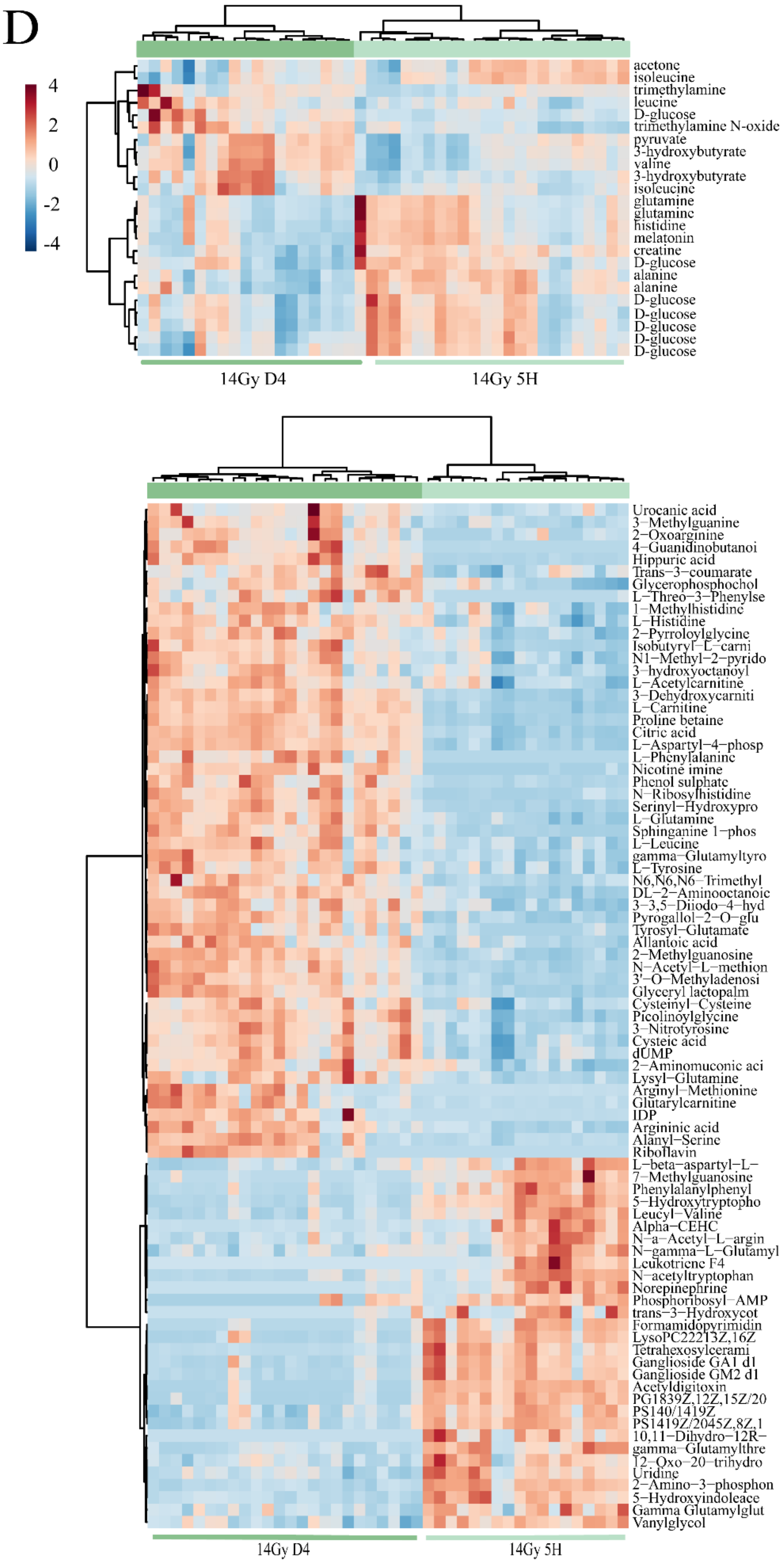

4
2
0
-2
-4
acetone
isoleucine
trimethylamine
leucine
D-glucose
trimethylamine N-oxide
pyruvate
3-hydroxybutyrate
valine
3-hydroxybutyrate
isoleucine
glutamine
glutamine
histidine
melatonin
creatine
D-glucose
alanine
alanine
D-glucose
D-glucose
D-glucose
D-glucose
D-glucose
14Gy D4
14Gy 5H
Urocanic acid
3−Methylguanine
2−Oxoarginine
4−Guanidinobutanoi
Hippuric acid
Trans−3−coumarate
Glycerophosphochol
L−Threo−3−Phenylse
1−Methylhistidine
L−Histidine
2−Pyrroloylglycine
Isobutyryl−L−carni
N1−Methyl−2−pyrido
3−hydroxyoctanoyl
L−Acetylcarnitine
3−Dehydroxycarniti
L−Carnitine
Proline betaine
Citric acid
L−Aspartyl−4−phosp
L−Phenylalanine
Nicotine imine
Phenol sulphate
N−Ribosylhistidine
Serinyl−Hydroxypro
L−Glutamine
Sphinganine 1−phos
L−Leucine
gamma−Glutamyltyro
L−Tyrosine
N6,N6,N6−Trimethyl
DL−2−Aminooctanoic
3−3,5−Diiodo−4−hyd
Pyrogallol−2−O−glu
Tyrosyl−Glutamate
Allantoic acid
2−Methylguanosine
N−Acetyl−L−methion
3'−O−Methyladenosi
Glyceryl lactopalm
Cysteinyl−Cysteine
Picolinoylglycine
3−Nitrotyrosine
Cysteic acid
dUMP
2−Aminomuconic aci
Lysyl−Glutamine
Arginyl−Methionine
Glutarylcarnitine
IDP
Argininic acid
Alanyl−Serine
Riboflavin
L−beta−aspartyl−L−
7−Methylguanosine
Phenylalanylphenyl
5−Hydroxytryptopho
Leucyl−Valine
Alpha−CEHC
N−a−Acetyl−L−argin
N−gamma−L−Glutamyl
Leukotriene F4
N−acetyltryptophan
Norepinephrine
Phosphoribosyl−AMP
trans−3−Hydroxycot
Formamidopyrimidin
LysoPC22213Z,16Z
Tetrahexosylcerami
Ganglioside GA1 d1
Ganglioside GM2 d1
Acetyldigitoxin
PG1839Z,12Z,15Z/20
PS140/1419Z
PS1419Z/2045Z,8Z,1
10,11−Dihydro−12R−
gamma−Glutamylthre
12−Oxo−20−trihydro
Uridine
2−Amino−3−phosphon
5−Hydroxyindoleace
Gamma Glutamylglut
Vanylglycol
14Gy D4
14Gy 5H

Figure S1. NMR and LC-MS time-dependent metabolomics analysis of cohort 1 for following 14 Gy ^{60}Co-γ-radiation exposure. **(A)** LC-MS metabolic trajectories of 14 Gy (*top*) and Sham (*bottom*) groups calculated by PLS (one Predictive Component (PC), Unit Variance (UV), 7-fold Cross Validation) from a baseline norm (5H) and visualized across time (Q^2). * indicates models p-value < 0.05 **(B)** Model analysis by SUS principles for significant effects as a result of radiation across two time points 14GyD4, 14GyD1 and a control ShD2 showing within and between group comparisons. **(C)** T v U scores plot (PLS - 1 Predictive Component, Unit Variance, 7-fold Cross Validation) comparing 14 Gy D4 to 5H baseline NMR data (*left*) and LC-MS data (*right*) and p-value v fold change volcano plot (LIMMA - moderated T-test, Benjamini-Hochberg correction) comparing 14 Gy D4 to 5H baseline NMR data (*left*) and LC-MS data (*right*) **(D)** Heatmap of NMR (*top*) LC-MS (*bottom*) mice serum for 14Gy D4 (vs 5H).

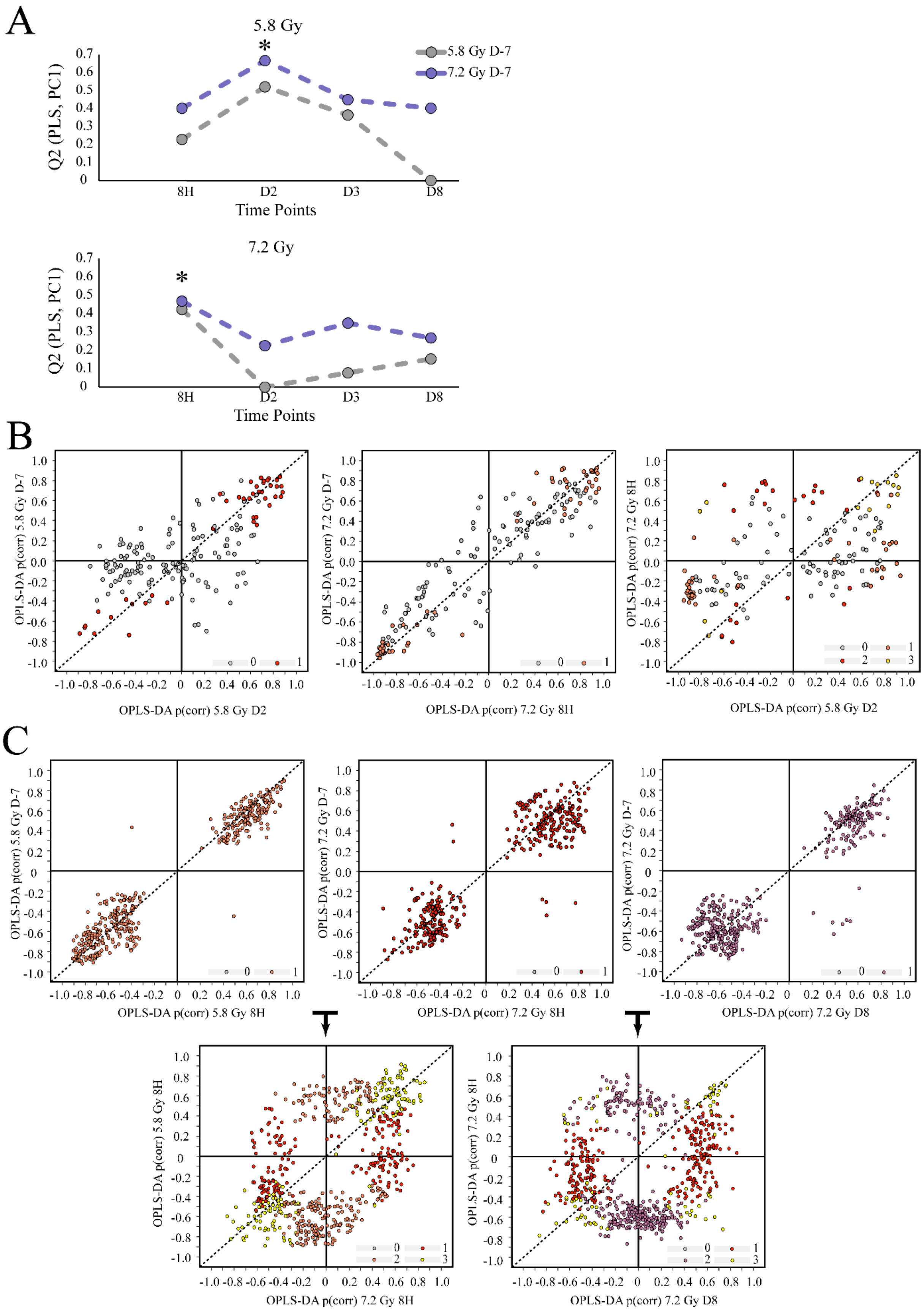
A
5.8 Gy
5.8 Gy D-7
7.2 Gy D-7
Q2 (PLS, PC1)
0.7
0.6
0.5
0.4
0.3
0.2
0.1
0
*
8H
D2
D3
D8
Time Points
7.2 Gy
Q2 (PLS, PC1)
*
8H
D2
D3
D8
Time Points
B
OPLS-DA p(corr) 5.8 Gy D-7
OPLS-DA p(corr) 5.8 Gy D2
OPLS-DA p(corr) 7.2 Gy D-7
OPLS-DA p(corr) 7.2 Gy 8H
OPLS-DA p(corr) 7.2 Gy 8H
OPLS-DA p(corr) 5.8 Gy D2
C
OPLS-DA p(corr) 5.8 Gy D-7
OPLS-DA p(corr) 5.8 Gy 8H
OPLS-DA p(corr) 7.2 Gy D-7
OPLS-DA p(corr) 7.2 Gy 8H
OPLS-DA p(corr) 7.2 Gy D-7
OPLS-DA p(corr) 7.2 Gy D8
OPLS-DA p(corr) 5.8 Gy 8H
OPLS-DA p(corr) 7.2 Gy 8H
OPLS-DA p(corr) 7.2 Gy 8H
OPLS-DA p(corr) 7.2 Gy D8

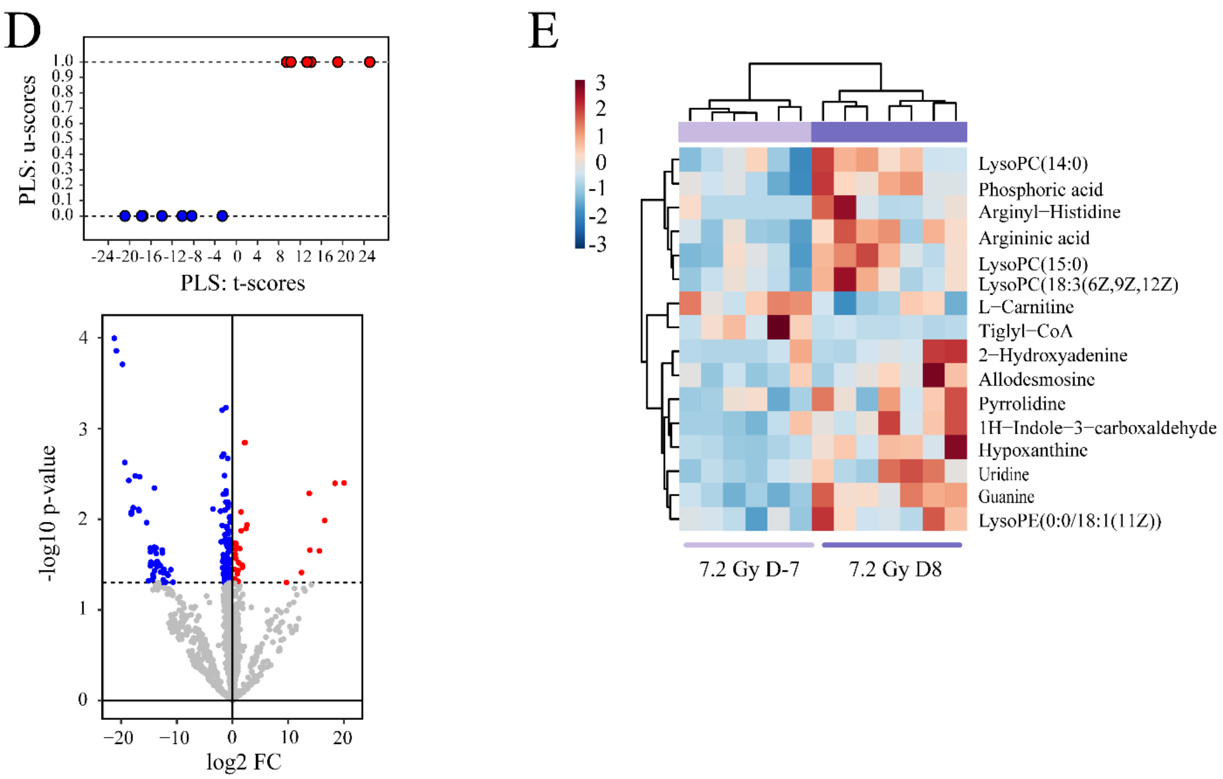

Figure S2. LC-MS time-dependent metabolomics analysis of cohort 2 samples following either 5.8 or 7.2 Gy ^{60}Co-γ-radiation exposure. **(A)** NMR metabolic trajectories from NHP serum after exposure to 5.8 Gy (*top*) or 7.2 Gy (*bottom*) radiation dose. Metabolic trajectories were calculated by PLS (one Predictive Component (PC), Unit Variance (UV), 7-fold Cross Validation) from a baseline norm (5H) and visualized across time (Q^2). * indicates models p-value < 0.05 **(B) NMR** model analysis by SUS principles for significant effects (VIP >1) as a result of radiation across within group 5.8 Gy D2 and 7.2 Gy 8H compared to respective controls and between group comparison of 5.8 Gy D2 and 7.2 Gy 8H. **(C)** LC-MS model analysis by SUS principles for significant effects (VIP > 1) as a result of radiation across within group comparisons 5.8 Gy 8H, 7.2 Gy 8H, and 7.2 Gy D8 compared to respective contorols and between group comparisons 5.8 Gy 8H vs 7.2 Gy 8H and 7.2 Gy 8H vs 7.2 Gy D8. **(D)** LC-MS T v U scores plot (*top*) (PLS - 1 Predictive Component, Unit Variance, 7-fold Cross Validation) comparing 7.2 Gy D8 to 7.2 Gy

D-7 baseline LC-MS data and a p-value versus fold change volcano plot (*bottom*) comparing LC-MS data 7.2 Gy D1 to D4 (LIMMA - moderated T-test, Benjamini-Hochberg correction). **(E)** Heatmap of putative LC-MS metabolite IDs comparing 7.2 Gy D-7 (light purple) to 7.2 Gy D8 (dark purple).

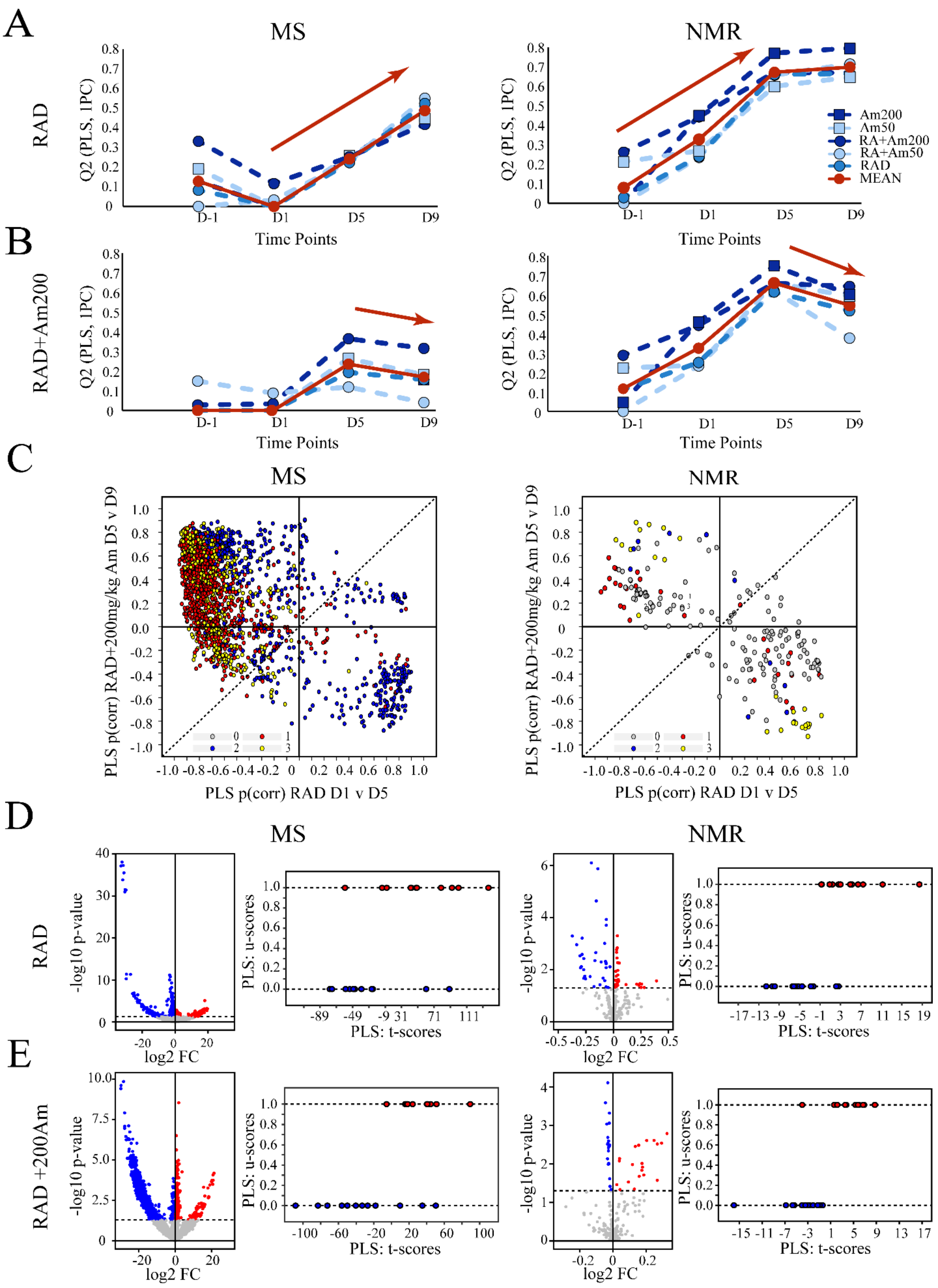
A
B
C
D
E
MS
NMR
RAD
RAD+Am200
RAD +200Am
Q2 (PLS, 1PC)
Time Points
D-1
D1
D5
D9
Am200
Am50
RA+Am200
RA+Am50
RAD
MEAN
PLS p(corr) RAD+200mg/kg Am D5 v D9
PLS p(corr) RAD D1 v D5
-log10 p-value
log2 FC
PLS: u-scores
PLS: t-scores

Figure S3. Combined ^{1}H NMR and LC-MS metabolomic analysis of cohort 3 exposed to 9.6 Gy ^{60}Co γ-radiation. **(A)** 0 mg/kg (RAD) and **(B)** 200 mg/kg (RAD_Am200) – trajectories calculated by PLS (1 Predictive Component (PC), Unit Variance (UV), 7-fold Cross Validation), from a baseline norm (D-5), visualized across time (Q2). **(C)** LC-MS (*left*) and NMR (*right*) model analysis by SUS principles for significant effects (VIP >1) as a result of radiation for between group comparison RAD D1 vs D5 and RAD+200mg/kg D5 vs D9 **(D-E)** LC-MS and NMR T v U scores plot (PLS - 1 Predictive Component, Unit Variance, 7-fold Cross Validation) and p-value v FC volcano plot (LIMMA - moderated T-test, Benjamini-Hochberg correction) for 9.6 Gy radiation exposure (RAD D1 vs D5) and 9.6 Gy radiation exposure with amifostine treatment at 200mg/kg (RAD+Am200 D5 vs D9).

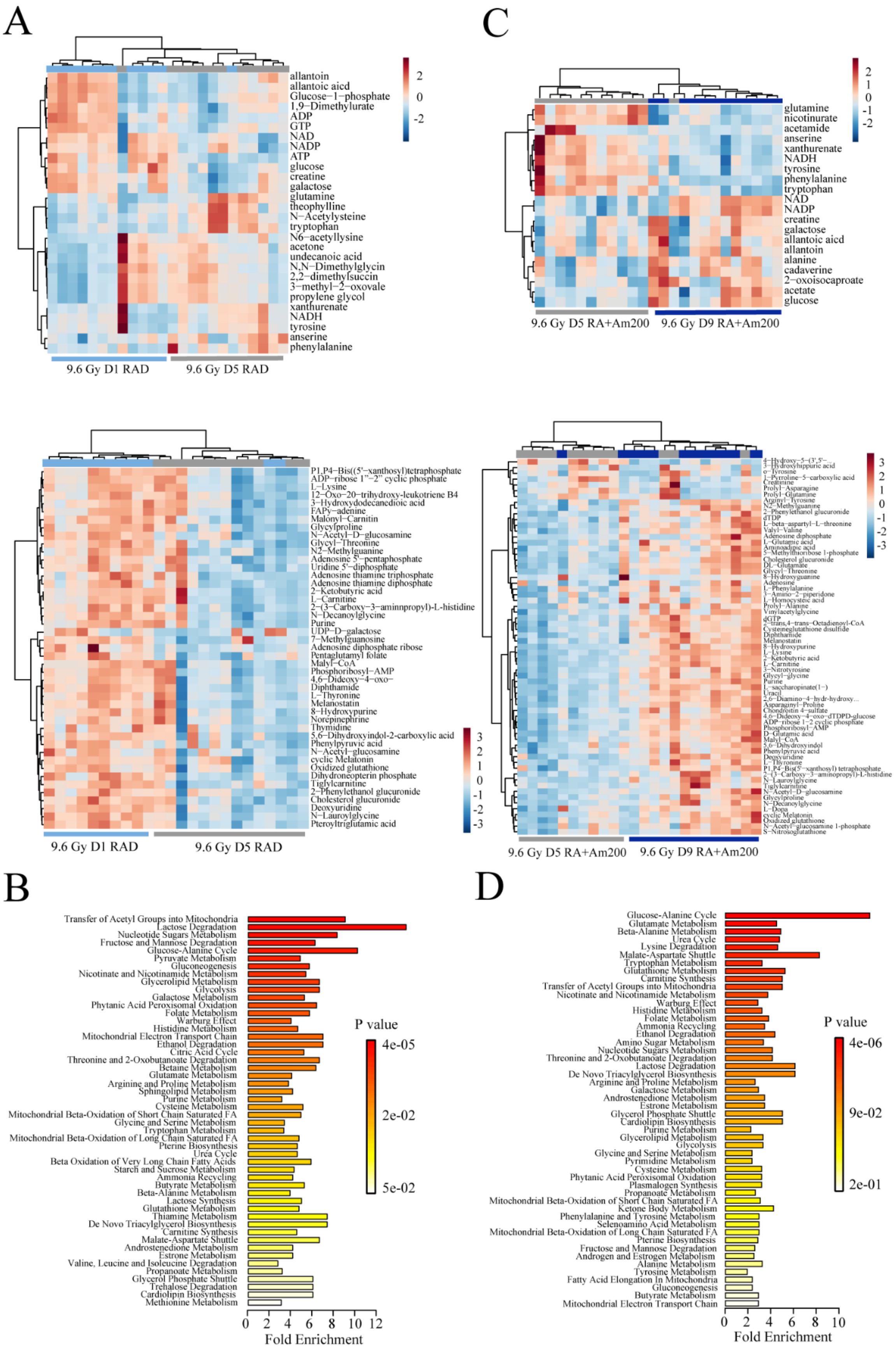
A
allantoin
allantoic aicd
Glucose−1−phosphate
1,9−Dimethylurate
ADP
GTP
NAD
NADP
ATP
glucose
creatine
galactose
glutamine
theophylline
N−Acetylysteine
tryptophan
N6−acetyllysine
acetone
undecanoic acid
N,N−Dimethylglycin
2,2−dimethylsuccin
3−methyl−2−oxovale
propylene glycol
xanthurenate
NADH
tyrosine
anserine
phenylalanine
9.6 Gy D1 RAD
9.6 Gy D5 RAD
C
glutamine
nicotinurate
acetamide
anserine
xanthurenate
NADH
tyrosine
phenylalanine
tryptophan
NAD
NADP
creatine
galactose
allantoic aicd
allantoin
alanine
cadaverine
2−oxoisocaproate
acetate
glucose
9.6 Gy D5 RA+Am200
9.6 Gy D9 RA+Am200
P1,P4−Bis((5'−xanthosyl)tetraphosphate
ADP−ribose 1''−2'' cyclic phosphate
L−Lysine
12−Oxo−20−trihydroxy−leukotriene B4
3−Hydroxydodecanedioic acid
FAPy−adenine
Malonyl−Carnitin
Glycylproline
N−Acetyl−D−glucosamine
Glycyl−Threonine
N2−Methylguanine
Adenosine 5'−pentaphosphate
Uridine 5'−diphosphate
Adenosine thiamine triphosphate
Adenosine thiamine diphosphate
2−Ketobutyric acid
L−Carnitine
2−(3−Carboxy−3−aminnpropyl)−L−histidine
N−Decanoylglycine
Purine
UDP−D−galactose
7−Methylguanosine
Adenosine diphosphate ribose
Pentaglutamyl folate
Malyl−CoA
Phosphoribosyl−AMP
4,6−Dideoxy−4−oxo−
Diphthamide
L−Thyronine
Melanostatin
8−Hydroxypurine
Norepinephrine
Thymidine
5,6−Dihydroxyindol-2-carboxylic acid
Phenylpyruvic acid
N−Acetyl−glucosamine
cyclic Melatonin
Oxidized glutathione
Dihydroneopterin phosphate
Tiglylcarnitine
2−Phenylethanol glucuronide
Cholesterol glucuronide
Deoxyuridine
N−Lauroylglycine
Pteroyltriglutamic acid
9.6 Gy D1 RAD
9.6 Gy D5 RAD
9.6 Gy D5 RA+Am200
9.6 Gy D9 RA+Am200
B
Transfer of Acetyl Groups into Mitochondria
Lactose Degradation
Nucleotide Sugars Metabolism
Fructose and Mannose Degradation
Glucose-Alanine Cycle
Pyruvate Metabolism
Gluconeogenesis
Nicotinate and Nicotinamide Metabolism
Glycerolipid Metabolism
Glycolysis
Galactose Metabolism
Phytanic Acid Peroxisomal Oxidation
Folate Metabolism
Warburg Effect
Histidine Metabolism
Mitochondrial Electron Transport Chain
Ethanol Degradation
Citric Acid Cycle
Threonine and 2-Oxobutanoate Degradation
Betaine Metabolism
Glutamate Metabolism
Arginine and Proline Metabolism
Sphingolipid Metabolism
Purine Metabolism
Cysteine Metabolism
Mitochondrial Beta-Oxidation of Short Chain Saturated FA
Glycine and Serine Metabolism
Tryptophan Metabolism
Mitochondrial Beta-Oxidation of Long Chain Saturated FA
Pterine Biosynthesis
Urea Cycle
Beta Oxidation of Very Long Chain Fatty Acids
Starch and Sucrose Metabolism
Ammonia Recycling
Butyrate Metabolism
Beta-Alanine Metabolism
Lactose Synthesis
Glutathione Metabolism
Thiamine Metabolism
De Novo Triacylglycerol Biosynthesis
Carnitine Synthesis
Malate-Aspartate Shuttle
Androstenedione Metabolism
Estrone Metabolism
Valine, Leucine and Isoleucine Degradation
Propanoate Metabolism
Glycerol Phosphate Shuttle
Trehalose Degradation
Cardiolipin Biosynthesis
Methionine Metabolism
P value
4e-05
2e-02
5e-02
0 2 4 6 8 10 12
Fold Enrichment
D
Glucose-Alanine Cycle
Glutamate Metabolism
Beta-Alanine Metabolism
Urea Cycle
Lysine Degradation
Malate-Aspartate Shuttle
Tryptophan Metabolism
Glutathione Metabolism
Carnitine Synthesis
Transfer of Acetyl Groups into Mitochondria
Nicotinate and Nicotinamide Metabolism
Warburg Effect
Histidine Metabolism
Folate Metabolism
Ammonia Recycling
Ethanol Degradation
Amino Sugar Metabolism
Nucleotide Sugars Metabolism
Threonine and 2-Oxobutanoate Degradation
Lactose Degradation
De Novo Triacylglycerol Biosynthesis
Arginine and Proline Metabolism
Galactose Metabolism
Androstenedione Metabolism
Estrone Metabolism
Glycerol Phosphate Shuttle
Cardiolipin Biosynthesis
Purine Metabolism
Glycerolipid Metabolism
Glycolysis
Glycine and Serine Metabolism
Pyrimidine Metabolism
Cysteine Metabolism
Phytanic Acid Peroxisomal Oxidation
Plasmalogen Synthesis
Propanoate Metabolism
Mitochondrial Beta-Oxidation of Short Chain Saturated FA
Ketone Body Metabolism
Phenylalanine and Tyrosine Metabolism
Selenoamino Acid Metabolism
Mitochondrial Beta-Oxidation of Long Chain Saturated FA
Pterine Biosynthesis
Fructose and Mannose Degradation
Androgen and Estrogen Metabolism
Alanine Metabolism
Tyrosine Metabolism
Fatty Acid Elongation In Mitochondria
Gluconeogenesis
Butyrate Metabolism
Mitochondrial Electron Transport Chain
P value
4e-06
9e-02
2e-01
0 2 4 6 8 10
Fold Enrichment

Figure S4. Combined ^{1}H NMR and LC-MS pathway analysis of cohort 3 exposed to 9.6 Gy ^{60}Co γ-radiation. **(A)** Heatmap of 9.6 Gy D1 (light blue) vs D5 (gray) NMR putative metabolite IDs (*top*) and LC-MS putative metabolite IDs (*bottom*). **(B)** MetaboAnalyst pathway enrichment analysis of NMR and LC-MS metabolites from (A). Top perturbed pathways are displayed by fold enrichment. **(C)** Heatmap of 9.6 Gy and 200mg/kg amifostine treatment D5 (gray) vs D9 (navy blue) NMR putative metabolite IDs (*top*) and LC-MS putative metabolite IDs (*bottom*). **(D)** MetaboAnalyst pathway enrichment analysis of NMR and LC-MS metabolites from (C). Top perturbed pathways are displayed by fold enrichment.

Table S1. Perturbed Metabolites of Cohort 1

Metabolite ID	VIP	pcorr	FC	t-score	p-value	FDR
10,11-Dihydro-12R-hydroxy-leukotriene E4	1.473	0.644	32.452	16.737	0.000	0.000
12-Oxo-20-trihydroxy-leukotriene B4	1.469	0.623	1.285	5.309	0.000	0.000
1-Methylhistidine	1.252	-0.669	-0.731	-4.878	0.000	0.000
2-Amino-3-phosphonopropionic acid	1.855	0.821	1.828	12.216	0.000	0.000
2-Aminomuconic acid semialdehyde	1.355	-0.749	-0.995	-6.156	0.000	0.000
2-Methylguanosine	1.710	-0.849	-1.448	-11.192	0.000	0.000
2-Oxoarginine	1.136	-0.399	-16.821	-4.754	0.000	0.000
2-Pyrroloylglycine	1.366	-0.782	-0.456	-5.297	0.000	0.000
3-(3,5-Diiodo-4-hydroxyphenyl)pyruvate	1.456	-0.740	-10.248	-3.425	0.001	0.004
3-Dehydroxycarnitine	1.914	-0.926	-1.830	-16.483	0.000	0.000
3-Hydroxybutyrate	2.248	0.548	0.763	6.037	0.000	0.000
3-hydroxyoctanoyl carnitine	1.252	-0.679	-1.011	-5.247	0.000	0.000
3-Nitrotyrosine	1.529	-0.841	-0.455	-6.481	0.000	0.000
3'-O-Methyladenosine	1.752	-0.852	-1.437	-12.991	0.000	0.000
4-Guanidinobutanoic acid	1.445	-0.607	-12.412	-5.403	0.000	0.000
5-Hydroxyindoleacetic acid	1.709	0.740	2.236	9.615	0.000	0.000
5-Hydroxytryptophol glucuronide	1.796	0.959	3.481	9.317	0.000	0.000
7-Methylguanine	1.391	-0.604	-1.542	-6.151	0.000	0.000
7-Methylguanosine	1.462	0.817	23.269	6.309	0.000	0.000
Acetyldigitoxin	1.818	0.922	28.724	7.150	0.000	0.000
Alanyl-Serine	1.539	-0.791	-1.199	-7.794	0.000	0.000
Allantoic acid	1.478	-0.775	-1.090	-7.711	0.000	0.000
Alpha-CEHC	1.282	0.753	2.840	5.057	0.000	0.000
Argininic acid	1.647	-0.770	-2.507	-8.848	0.000	0.000
Arginyl-Methionine	1.167	-0.628	-16.887	-3.968	0.001	0.001
Citric acid	1.841	-0.893	-1.570	-13.256	0.000	0.000
Creatine	1.806	-0.759	-0.876	-4.674	0.001	0.012
Cysteic acid	1.543	-0.836	-0.593	-6.901	0.000	0.000
Cysteinyl-Cysteine	1.322	-0.751	-0.423	-4.998	0.000	0.000
D-Glucose	1.810	0.477	0.773	5.433	0.000	0.001
DL-2-Aminooctanoic acid	1.671	-0.805	-1.162	-10.025	0.000	0.000
dUMP	1.447	-0.792	-0.540	-6.096	0.000	0.000
Formamidopyrimidine nucleoside triphosphate	1.510	0.782	28.280	7.894	0.000	0.000
Gamma Glutamylglutamic acid	1.279	0.582	1.100	3.916	0.001	0.003
gamma-Glutamylthreonine	1.578	0.670	0.917	7.784	0.000	0.000
Gamma-Glutamyltyrosine	1.574	-0.726	-1.156	-8.747	0.000	0.000
Ganglioside GA1 (d18:1/16:0)	1.517	0.771	4.157	4.865	0.006	0.012
Ganglioside GM2 (d18:1/18:1(11Z))	1.676	0.807	2.856	8.984	0.000	0.000
Glutaminyl-Lysine	1.398	-0.694	-3.651	-2.398	0.021	0.039
Glutarylcarnitine	1.310	-0.700	-10.734	-2.780	0.011	0.022
Glycerophosphocholine	1.448	-0.776	-0.882	-6.192	0.000	0.000
Glyceryl lactopalmitate	1.712	-0.865	-2.017	-11.928	0.000	0.000
Hippuric acid	1.665	-0.721	-17.779	-5.700	0.000	0.000
IDP	1.223	-0.650	-6.609	-4.149	0.000	0.001
L-Acetylcarnitine	1.216	-0.701	-0.379	-4.112	0.000	0.001
L-Alanine	1.890	-0.626	-0.385	-4.250	0.002	0.014
L-Aspartyl-4-phosphate	1.901	-0.914	-0.964	-14.119	0.000	0.000
L-beta-aspartyl-L-threonine	1.434	0.658	0.458	5.678	0.000	0.000
L-Carnitine	1.935	-0.952	-1.630	-17.887	0.000	0.000
Leucyl-Valine	1.498	0.855	0.917	7.046	0.000	0.000
Leukotriene F4	1.205	0.648	24.627	6.666	0.000	0.000
L-Glutamine	1.199	-0.759	-1.343	-3.797	0.001	0.010
L-Glutamine	1.563	-0.709	-1.605	-6.100	0.000	0.000
L-Histidine	1.524	-0.784	-0.765	-6.539	0.000	0.000
L-Histidine	1.205	-0.781	-1.565	-3.919	0.001	0.011
L-Isoleucine	1.185	-0.511	-0.586	-4.436	0.002	0.005
L-Isoleucine	1.588	0.278	0.479	3.175	0.006	0.022
L-Leucine	1.860	0.416	0.399	4.214	0.001	0.004
L-Phenylalanine	1.536	-0.698	-30.810	-19.686	0.000	0.000

L-Tyrosine	1.267	-0.575	-7.322	-2.574	0.021	0.038
LysoPC(22:2(13Z,16Z))	1.357	0.742	5.983	4.935	0.002	0.005
Melatonin	1.198	-0.793	-1.448	-3.675	0.005	0.031
N1-Methyl-2-pyridone-5-carboxamide	1.408	-0.734	-0.774	-5.873	0.000	0.000
N6,N6,N6-Trimethyl-L-lysine	1.369	-0.647	-4.181	-2.743	0.012	0.023
N-a-Acetyl-L-arginine	1.139	0.712	1.298	4.614	0.000	0.000
N-Acetyl-L-methionine	1.767	-0.848	-2.431	-13.519	0.000	0.000
N-acetyltryptophan	1.376	0.785	1.483	5.892	0.000	0.000
N-gamma-L-Glutamyl-D-alanine	1.145	0.690	1.232	3.285	0.002	0.005
Nicotine imine	1.763	-0.797	-27.502	-11.236	0.000	0.000
N-Ribosylhistidine	1.640	-0.710	-16.569	-4.893	0.000	0.000
O-isobutyryl-L-carnitine	1.449	-0.705	-0.978	-7.048	0.000	0.000
PG(18:3(9Z,12Z,15Z)/20:3(5Z,8Z,11Z))	1.791	0.913	20.073	5.208	0.000	0.000
Phenol sulphate	1.536	-0.785	-6.519	-3.174	0.003	0.007
Phenylalanylphenylalanine	1.457	0.823	3.120	7.195	0.000	0.000
Phenylpyruvic acid	1.296	-0.569	-22.132	-6.737	0.000	0.000
Phosphoribosyl-AMP	1.247	0.649	21.065	8.395	0.000	0.000
p-Hydroxyphenylacetic acid	1.235	0.689	38.628	6.468	0.000	0.000
Picolinoylglycine	1.783	-0.935	-1.108	-10.522	0.000	0.000
Proline betaine	1.924	-0.913	-1.607	-19.221	0.000	0.000
PS(14:0/14:1(9Z))	1.336	0.743	1.464	5.152	0.000	0.000
PS(20:4(5Z,8Z,11Z,14Z)/14:1(9Z))	1.580	0.855	2.828	6.092	0.000	0.000
Pyrogallol-2-O-glucuronide	1.663	-0.736	-1.152	-9.817	0.000	0.000
Pyruvate	1.749	0.725	0.684	2.655	0.011	0.050
Riboflavin	1.467	-0.647	-25.009	-8.450	0.000	0.000
Serinyl-Hydroxyproline	1.709	-0.751	-2.468	-13.042	0.000	0.000
Sphinganine 1-phosphate	1.701	-0.803	-16.537	-5.334	0.000	0.000
Tetrahexosylceramide (d18:1/16:0)	1.612	0.768	9.423	3.798	0.000	0.001
Trans-3-coumarate	1.158	-0.487	-0.432	-4.460	0.000	0.001
trans-3-Hydroxycotinine glucuronide	1.074	0.562	16.821	3.841	0.000	0.001
Trimethylamine N-oxide	2.226	0.408	0.759	5.884	0.000	0.000
Tyrosyl-Glutamate	1.440	-0.722	-20.510	-6.076	0.000	0.000
Uridine	1.488	0.643	0.782	6.354	0.000	0.000
Urocanic acid	1.261	-0.500	-1.633	-7.595	0.000	0.000
Valine	2.354	0.784	0.831	5.198	0.000	0.000
Vanylglycol	1.386	0.741	0.670	4.958	0.001	0.002

Table S2. Perturbed Pathways of Cohort 1.

Enriched Pathways	FC	p-value	FDR
Methylhistidine Metabolism	0.250	0.299	1
Glucose-Alanine Cycle	0.231	0.0909	1
Transfer of Acetyl Groups into Mitochondria	0.136	0.285	1
Ammonia Recycling	0.125	0.286	1
Beta Oxidation of Very Long Chain Fatty Acids	0.118	0.431	1
Alanine Metabolism	0.118	0.431	1
Phenylacetate Metabolism	0.111	0.552	1
Lactose Degradation	0.111	0.552	1
Phenylalanine and Tyrosine Metabolism	0.107	0.43	1
Urea Cycle	0.103	0.454	1
Pyruvaldehyde Degradation	0.100	0.59	1
Carnitine Synthesis	0.091	0.571	1
D-Arginine and D-Ornithine Metabolism	0.091	0.625	1
Taurine and Hypotaurine Metabolism	0.083	0.658	1
Glycolysis	0.080	0.643	1
Oxidation of Branched Chain Fatty Acids	0.077	0.664	1
Thyroid hormone synthesis	0.077	0.687	1
Histidine Metabolism	0.070	0.725	1
Warburg Effect	0.069	0.745	1
Tryptophan Metabolism	0.067	0.769	1
Citric Acid Cycle	0.063	0.773	1
Glutamate Metabolism	0.061	0.805	1
Amino Sugar Metabolism	0.061	0.788	1
Gluconeogenesis	0.057	0.815	1
Nicotinate and Nicotinamide Metabolism	0.054	0.839	1
Glycine and Serine Metabolism	0.051	0.894	1
Pyrimidine Metabolism	0.051	0.894	1
Catecholamine Biosynthesis	0.050	0.834	1
Riboflavin Metabolism	0.050	0.834	1
Lactose Synthesis	0.050	0.834	1
Sphingolipid Metabolism	0.050	0.87	1
Valine, Leucine and Isoleucine Degradation	0.050	0.901	1
Glutathione Metabolism	0.048	0.848	1
Tyrosine Metabolism	0.042	0.956	1
Cysteine Metabolism	0.038	0.904	1
Mitochondrial Beta-Oxidation of Short Chain Saturated Fatty Acids	0.037	0.912	1
Selenoamino Acid Metabolism	0.036	0.92	1
Mitochondrial Beta-Oxidation of Long Chain Saturated Fatty Acids	0.036	0.92	1
Pterine Biosynthesis	0.034	0.927	1
Beta-Alanine Metabolism	0.029	0.954	1
Aspartate Metabolism	0.029	0.958	1
Fatty Acid Biosynthesis	0.029	0.958	1
Retinol Metabolism	0.027	0.965	1
Purine Metabolism	0.027	0.991	1
Galactose Metabolism	0.026	0.968	1
Propanoate Metabolism	0.024	0.978	1
Fatty acid Metabolism	0.023	0.98	1
Pyruvate Metabolism	0.021	0.987	1
Arginine and Proline Metabolism	0.019	0.992	1

Table S3. Perturbed Metabolites of Cohort 2[a]

Metabolite ID	VIP[b]	Pcorr[c]	FC[d]	t-score[e]	p-value[f]	FDR[g]
1H-Indole-3-carboxaldehyde	1.871	-0.424	-1.956	-2.710	0.019	0.461
2-Hydroxyadenine	1.236	-0.260	-14.061	-2.587	0.025	0.467
Allodesmosine	1.653	-0.548	-1.627	-2.651	0.042	0.433
Argininic acid	2.346	-0.785	-1.287	-3.826	0.004	0.317
Arginyl-Histidine	1.366	-0.557	-15.196	-2.707	0.027	0.476
Guanine	2.512	-0.790	-3.657	-3.330	0.006	0.351
Hypoxanthine	2.197	-0.659	-1.774	-4.665	0.001	0.208
L-Carnitine	2.242	0.757	0.818	3.499	0.014	0.380
LysoPC(14:0)	1.995	-0.601	-0.908	-2.720	0.019	0.452
LysoPC(15:0)	2.162	-0.673	-0.938	-3.143	0.009	0.386
LysoPC(18:3(6Z,9Z,12Z))	1.827	-0.664	-0.664	-2.427	0.033	0.489
LysoPE(0:0/18:1(11Z))	1.828	-0.744	-1.087	-2.326	0.039	0.516
Phosphoric acid	1.879	-0.653	-0.628	-2.434	0.041	0.566
Pyrrolidine	1.837	-0.616	-0.407	-2.271	0.047	0.499
Tiglyl-CoA	1.658	0.519	2.298	3.386	0.012	0.398
Uridine	1.858	-0.437	-0.778	-2.498	0.028	0.510

Table S4. Perturbed Pathways of Cohort 2.

Enriched Pathways	FC	p-value	FDR
Glycerol Phosphate Shuttle	0.091	0.160	1.000
Beta Oxidation of Very Long Chain Fatty Acids	0.059	0.237	1.000
Vitamin B6 Metabolism	0.050	0.272	1.000
Lactose Synthesis	0.050	0.272	1.000
Glutathione Metabolism	0.048	0.284	1.000
Carnitine Synthesis	0.045	0.295	1.000
Purine Metabolism	0.041	0.103	1.000
Glycolysis	0.040	0.329	1.000
Cysteine Metabolism	0.038	0.339	1.000
Oxidation of Branched Chain Fatty Acids	0.038	0.339	1.000
Inositol Phosphate Metabolism	0.038	0.339	1.000
Mitochondrial Beta-Oxidation of Short Chain Saturated Fatty Acids	0.037	0.350	1.000
Selenoamino Acid Metabolism	0.036	0.360	1.000
Mitochondrial Beta-Oxidation of Long Chain Saturated Fatty Acids	0.036	0.360	1.000
Urea Cycle	0.034	0.371	1.000
Pyrimidine Metabolism	0.034	0.234	1.000
Ammonia Recycling	0.031	0.401	1.000
Fructose and Mannose Degradation	0.031	0.401	1.000
Inositol Metabolism	0.030	0.410	1.000
Gluconeogenesis	0.029	0.429	1.000
Nicotinate and Nicotinamide Metabolism	0.027	0.447	1.000
Propanoate Metabolism	0.024	0.491	1.000
Fatty acid Metabolism	0.023	0.499	1.000
Pyruvate Metabolism	0.021	0.539	1.000
Glutamate Metabolism	0.020	0.546	1.000
Arginine and Proline Metabolism	0.019	0.575	1.000
Warburg Effect	0.017	0.609	1.000
Valine, Leucine and Isoleucine Degradation	0.017	0.622	1.000
Arachidonic Acid Metabolism	0.014	0.675	1.000

Table S5. Perturbed Metabolites of Cohort 3 RAD D1vD5

Metabolite ID	VIP	Pcorr	FC	t-score	p-value	FDR
1,9-Dimethylurate	1.332	-0.796	-0.269	-2.470	0.021	0.115
2-(3-Carboxy-3-aminopropyl)-L-Histidine	2.038	-0.786	-0.754	-3.192	0.004	0.277
2,2-Dimethylsuccinic acid	1.201	0.796	0.392	2.361	0.027	0.120
3-Methyl-2-oxovalerate	1.129	0.794	0.211	2.228	0.036	0.144
Adenosine diphosphate ribose	1.697	-0.503	-9.139	-2.202	0.038	0.615
Adenosine thiamine triphosphate	1.429	-0.695	-0.705	-2.308	0.030	0.578
ADP	2.269	-0.920	-0.142	-6.455	0.000	0.000
Allantoic acid	1.540	-0.808	-0.111	-3.098	0.005	0.047
Anserine	1.990	0.799	0.036	4.045	0.000	0.015
ATP	2.269	-0.767	-0.200	-6.678	0.000	0.000
Cholesterol glucuronide	1.866	-0.888	-0.586	-2.525	0.019	0.520
Creatine	1.515	-0.714	-0.292	-2.922	0.008	0.059
D-Galactose	1.550	-0.832	-0.169	-3.147	0.004	0.047
D-Glucose	1.735	-0.641	-0.334	-3.728	0.001	0.022
GTP	1.549	-0.884	-0.068	-3.083	0.005	0.047
L-Carnitine	1.160	-0.659	-0.535	-2.102	0.047	0.617
L-Phenylalanine	1.823	0.375	0.035	3.613	0.001	0.027
L-Tryptophan	1.887	0.708	0.029	3.548	0.002	0.029
L-Tryptophan	1.668	0.492	0.021	2.740	0.012	0.074
Malonyl-Carnitin	1.690	-0.912	-0.438	-2.192	0.039	0.617
N,N-Dimethylglycine	1.046	0.776	0.280	2.086	0.048	0.153
N6-acetyllysine	1.115	0.749	0.114	2.212	0.037	0.144
N-Acetyl-D-glucosamine	2.142	-0.878	-0.874	-3.069	0.005	0.317
N-Acetylysteine	1.375	0.569	0.024	2.369	0.027	0.120
NAD+	1.965	-0.660	-0.065	-4.418	0.000	0.008
NADH	1.556	-0.836	-0.072	-3.119	0.005	0.047
NADP+	2.010	-0.638	-0.064	-4.622	0.000	0.006
N-Decanoylglycine	1.729	-0.811	-0.639	-2.370	0.027	0.567
Phenylpyruvic acid	1.620	-0.758	-0.735	-2.616	0.015	0.487
Phosphoribosyl-AMP	1.407	-0.887	-0.502	-2.124	0.045	0.617
Propylene glycol	1.062	0.787	0.252	2.084	0.048	0.153
Purine	1.697	-0.765	-0.501	-2.437	0.023	0.550
Tiglylcarnitine	2.386	-0.754	-1.848	-2.817	0.010	0.412
Undecanoic acid	1.101	0.789	0.256	2.229	0.036	0.144
Xanthurenate	1.362	0.712	0.040	2.598	0.016	0.100

Table S6. Perturbed Pathways of Cohort 3 RAD D1vD5

Enriched Pathways	FC	p-value	FDR
Lactose Degradation	0.444	0.000	0.006
Glucose-Alanine Cycle	0.308	0.001	0.010
Transfer of Acetyl Groups into Mitochondria	0.273	0.000	0.004
Nucleotide Sugars Metabolism	0.250	0.000	0.010
Thiamine Metabolism	0.222	0.033	0.080
De Novo Triacylglycerol Biosynthesis	0.222	0.033	0.080
Mitochondrial Electron Transport Chain	0.211	0.003	0.016
Ethanol Degradation	0.211	0.003	0.016
Glycerolipid Metabolism	0.200	0.001	0.010
Glycolysis	0.200	0.001	0.010
Threonine and 2-Oxobutanoate Degradation	0.200	0.003	0.017
Malate-Aspartate Shuttle	0.200	0.041	0.094
Phytanic Acid Peroxisomal Oxidation	0.192	0.001	0.010
Betaine Metabolism	0.190	0.004	0.020
Fructose and Mannose Degradation	0.188	0.000	0.010
Glycerol Phosphate Shuttle	0.182	0.049	0.094
Trehalose Degradation	0.182	0.049	0.094
Cardiolipin Biosynthesis	0.182	0.049	0.094
Beta Oxidation of Very Long Chain Fatty Acids	0.176	0.017	0.050
Folate Metabolism	0.172	0.002	0.014
Gluconeogenesis	0.171	0.001	0.010
Phosphatidylethanolamine Biosynthesis	0.167	0.057	0.100
Nicotinate and Nicotinamide Metabolism	0.162	0.001	0.010
Galactose Metabolism	0.158	0.001	0.010
Butyrate Metabolism	0.158	0.023	0.063
Citric Acid Cycle	0.156	0.003	0.017
Cysteine Metabolism	0.154	0.009	0.035
Ketone Body Metabolism	0.154	0.066	0.108
Lactose Synthesis	0.150	0.026	0.069
Mitochondrial Beta-Oxidation of Short Chain Saturated Fatty Acids	0.148	0.010	0.039
Pyruvate Metabolism	0.146	0.001	0.010
Mitochondrial Beta-Oxidation of Long Chain Saturated Fatty Acids	0.143	0.012	0.040
Glutathione Metabolism	0.143	0.030	0.076
Phosphatidylcholine Biosynthesis	0.143	0.076	0.118
Histidine Metabolism	0.140	0.002	0.014
Pterine Biosynthesis	0.138	0.013	0.042
Urea Cycle	0.138	0.013	0.042
Carnitine Synthesis	0.136	0.034	0.080
Starch and Sucrose Metabolism	0.129	0.017	0.050
Sphingolipid Metabolism	0.125	0.009	0.035
Ammonia Recycling	0.125	0.019	0.054
Androstenedione Metabolism	0.125	0.042	0.094
Estrone Metabolism	0.125	0.042	0.094
Biotin Metabolism	0.125	0.237	0.302
Glutamate Metabolism	0.122	0.004	0.020
Warburg Effect	0.121	0.002	0.014
Beta-Alanine Metabolism	0.118	0.023	0.063
Alanine Metabolism	0.118	0.107	0.151
Phosphatidylinositol Phosphate Metabolism	0.118	0.107	0.151
Oxidation of Branched Chain Fatty Acids	0.115	0.052	0.094
Inositol Phosphate Metabolism	0.115	0.052	0.094
Plasmalogen Synthesis	0.115	0.052	0.094
Arginine and Proline Metabolism	0.113	0.006	0.028
Mitochondrial Beta-Oxidation of Medium Chain Saturated Fatty Acids	0.111	0.057	0.100
Phenylacetate Metabolism	0.111	0.263	0.326
Phenylalanine and Tyrosine Metabolism	0.107	0.062	0.103
Selenoamino Acid Metabolism	0.107	0.062	0.103
Pentose Phosphate Pathway	0.103	0.068	0.109
Glycine and Serine Metabolism	0.102	0.011	0.039
Tryptophan Metabolism	0.100	0.012	0.040

Lysine Degradation	0.100	0.074	0.116
Riboflavin Metabolism	0.100	0.140	0.193
Propanoate Metabolism	0.095	0.046	0.094
Pantothenate and CoA Biosynthesis	0.095	0.152	0.207
Purine Metabolism	0.095	0.009	0.035
Methionine Metabolism	0.093	0.050	0.094
Fatty acid Metabolism	0.093	0.050	0.094
Inositol Metabolism	0.091	0.093	0.137
Amino Sugar Metabolism	0.091	0.093	0.137
Androgen and Estrogen Metabolism	0.091	0.093	0.137
Sulfate/Sulfite Metabolism	0.091	0.164	0.220
Degradation of Superoxides	0.091	0.312	0.377
Fatty Acid Elongation In Mitochondria	0.086	0.106	0.151
Valine, Leucine and Isoleucine Degradation	0.083	0.044	0.094
Caffeine Metabolism	0.083	0.188	0.245
Retinol Metabolism	0.081	0.121	0.169
Bile Acid Biosynthesis	0.077	0.059	0.101
Vitamin K Metabolism	0.071	0.379	0.447
Steroidogenesis	0.070	0.168	0.222
Phospholipid Biosynthesis	0.069	0.250	0.314
Steroid Biosynthesis	0.063	0.210	0.271
Aspartate Metabolism	0.057	0.325	0.389
Spermidine and Spermine Biosynthesis	0.056	0.458	0.522
Pyrimidine Metabolism	0.051	0.311	0.377
Porphyrin Metabolism	0.050	0.387	0.452
Ubiquinone Biosynthesis	0.050	0.494	0.557
Tyrosine Metabolism	0.042	0.433	0.499
Arachidonic Acid Metabolism	0.014	0.910	1.000

Table S7. Perturbed Metabolites of Cohort 3 RAD+200 D5vD9

Metabolite ID	VIP	Pcorr	FC	t-score	p-value	FDR
1-Pyrroline-5-carboxylic acid	1.929	-0.062	-0.665	-3.239	0.004	0.058
2,6-Diamino-4-hydroxy-5-N-methylformamidopyrimidine	2.086	0.890	0.556	3.389	0.003	0.050
2-Ketobutyric acid	1.745	0.825	0.470	2.904	0.008	0.092
3-Nitrotyrosine	1.998	0.853	0.660	3.412	0.002	0.048
4,6-Dideoxy-4-oxo-dTDP-D-glucose	1.853	0.846	0.622	3.120	0.005	0.068
8-Hydroxyguanine	1.218	0.336	0.651	2.261	0.034	0.188
8-Hydroxypurine	1.292	0.812	0.363	2.237	0.035	0.192
Acetamide	1.493	-0.499	-0.018	-2.199	0.038	0.196
ADP-ribose 1"-2" cyclic phosphate	1.625	0.838	0.557	2.581	0.017	0.130
Anserine	1.927	-0.856	-0.033	-3.310	0.003	0.036
Arginyl-Tyrosine	1.941	0.425	0.566	2.098	0.047	0.216
Asparaginyl-Proline	1.647	0.853	0.576	2.880	0.009	0.095
Chondroitin 4-sulfate	1.459	0.691	0.402	2.217	0.037	0.195
Creatinine	1.602	0.091	-0.794	-2.770	0.011	0.106
Deoxyuridine	1.848	0.522	0.484	2.863	0.009	0.097
D-Galactose	1.551	0.734	0.159	2.479	0.021	0.123
D-Glucose	2.005	0.864	0.326	3.570	0.002	0.036
D-Glutamic acid	1.839	0.865	0.618	2.967	0.007	0.085
dGTP	1.353	0.738	0.636	2.508	0.020	0.143
DL-Glutamate	1.601	0.686	0.405	2.273	0.033	0.186
dTDP	1.548	0.667	0.522	2.544	0.018	0.136
Glycyl-glycine	1.882	0.837	0.722	3.109	0.005	0.069
Glycyl-Threonine	1.432	0.650	0.371	2.109	0.046	0.214
L-Alanine	1.628	0.284	0.118	2.791	0.010	0.069
L-Carnitine	1.664	0.769	0.474	2.745	0.012	0.108
L-Glutamic acid	1.716	0.618	0.477	2.529	0.019	0.139
L-Glutamine	2.509	-0.719	-0.031	-4.792	0.000	0.016
L-Lysine	1.552	0.744	0.395	2.404	0.025	0.163
L-Lysine	1.828	0.391	0.135	3.253	0.004	0.036
L-Phenylalanine	2.178	0.756	0.649	3.709	0.001	0.031
L-Phenylalanine	2.236	-0.724	-0.045	-4.313	0.000	0.026
L-saccharopinate(1-)	1.806	0.828	0.462	3.017	0.006	0.080
L-Thyronine	1.507	0.707	0.466	2.702	0.013	0.112
L-Tryptophan	2.491	-0.875	-0.022	-4.066	0.000	0.033
L-Tyrosine	1.933	-0.812	-0.028	-3.216	0.004	0.036
Malyl-CoA	1.149	0.797	0.580	2.196	0.039	0.199
N-Acetyl-D-glucosamine	1.616	0.698	0.520	2.676	0.014	0.116
N-Acetyl-glucosamine 1-phosphate	1.269	0.531	0.880	2.363	0.027	0.169
NAD+	1.755	0.461	0.040	2.947	0.007	0.060
NADH	1.770	-0.719	-0.025	-2.810	0.010	0.069
NADP+	1.733	0.401	0.039	2.887	0.008	0.066
N-Decanoylglycine	1.388	0.587	0.466	2.175	0.040	0.202
N-Lauroylglycine	2.069	0.546	0.853	3.503	0.002	0.041
o-Tyrosine	1.953	-0.185	-1.154	-3.642	0.001	0.034
Oxidized glutathione	1.805	0.774	0.570	2.763	0.011	0.107
Phosphoribosyl-AMP	1.631	0.837	0.530	2.657	0.014	0.119
Prolyl-Alanine	1.386	0.627	0.327	2.230	0.036	0.193
Prolyl-Asparagine	2.082	-0.003	-0.759	-3.708	0.001	0.031
Purine	2.126	0.830	0.545	3.488	0.002	0.043
Tiglylcarnitine	1.657	0.582	0.609	2.206	0.038	0.197
Uracil	2.050	0.824	0.666	3.549	0.002	0.039
Xanthurenate	2.024	-0.846	-0.026	-3.387	0.003	0.036

Table S8. Perturbed Pathways of Cohort 3 RAD+200 D5vD9

Enriched Pathways	FC	p-value	FDR
Glucose-Alanine Cycle	0.462	0.000	0.000
Malate-Aspartate Shuttle	0.300	0.006	0.094
Lactose Degradation	0.222	0.047	0.220
De Novo Triacylglycerol Biosynthesis	0.222	0.047	0.220
Glutathione Metabolism	0.190	0.008	0.094
Carnitine Synthesis	0.182	0.010	0.094
Transfer of Acetyl Groups into Mitochondria	0.182	0.010	0.094
Glycerol Phosphate Shuttle	0.182	0.068	0.224
Cardiolipin Biosynthesis	0.182	0.068	0.224
Beta-Alanine Metabolism	0.176	0.002	0.054
Urea Cycle	0.172	0.005	0.094
Lysine Degradation	0.167	0.005	0.094
Glutamate Metabolism	0.163	0.000	0.022
Ethanol Degradation	0.158	0.037	0.218
Ketone Body Metabolism	0.154	0.092	0.233
Nucleotide Sugars Metabolism	0.150	0.042	0.218
Threonine and 2-Oxobutanoate Degradation	0.150	0.042	0.218
Folate Metabolism	0.138	0.025	0.178
Nicotinate and Nicotinamide Metabolism	0.135	0.014	0.121
Ammonia Recycling	0.125	0.035	0.218
Androstenedione Metabolism	0.125	0.067	0.224
Estrone Metabolism	0.125	0.067	0.224
Biotin Metabolism	0.125	0.280	0.473
Amino Sugar Metabolism	0.121	0.039	0.218
Glycerolipid Metabolism	0.120	0.074	0.224
Glycolysis	0.120	0.074	0.224
Alanine Metabolism	0.118	0.146	0.317
Tryptophan Metabolism	0.117	0.008	0.094
Histidine Metabolism	0.116	0.025	0.178
Cysteine Metabolism	0.115	0.082	0.224
Phytanic Acid Peroxisomal Oxidation	0.115	0.082	0.224
Plasmalogen Synthesis	0.115	0.082	0.224
Mitochondrial Beta-Oxidation of Short Chain Saturated Fatty Acids	0.111	0.090	0.233
Phenylacetate Metabolism	0.111	0.309	0.488
Homocysteine Degradation	0.111	0.309	0.488
Phenylalanine and Tyrosine Metabolism	0.107	0.098	0.233
Selenoamino Acid Metabolism	0.107	0.098	0.233
Mitochondrial Beta-Oxidation of Long Chain Saturated Fatty Acids	0.107	0.098	0.233
Galactose Metabolism	0.105	0.061	0.224
Butyrate Metabolism	0.105	0.174	0.342
Mitochondrial Electron Transport Chain	0.105	0.174	0.342
Warburg Effect	0.103	0.024	0.178
Pterine Biosynthesis	0.103	0.106	0.247
Propanoate Metabolism	0.095	0.082	0.224
Betaine Metabolism	0.095	0.204	0.384
Arginine and Proline Metabolism	0.094	0.056	0.224
Fructose and Mannose Degradation	0.094	0.132	0.302
Androgen and Estrogen Metabolism	0.091	0.142	0.316
Degradation of Superoxides	0.091	0.363	0.548
Fatty Acid Elongation In Mitochondria	0.086	0.161	0.329
Gluconeogenesis	0.086	0.161	0.329
Glycine and Serine Metabolism	0.085	0.081	0.224
Pyrimidine Metabolism	0.085	0.081	0.224
Caffeine Metabolism	0.083	0.249	0.428
Purine Metabolism	0.081	0.069	0.224
Retinol Metabolism	0.081	0.181	0.348
Thyroid hormone synthesis	0.077	0.414	0.597
Sphingolipid Metabolism	0.075	0.213	0.393
Mitochondrial Beta-Oxidation of Medium Chain Saturated Fatty Acids	0.074	0.295	0.488
Vitamin K Metabolism	0.071	0.438	0.613

Methionine Metabolism	0.070	0.245	0.428
Fatty acid Metabolism	0.070	0.245	0.428
Steroidogenesis	0.070	0.245	0.428
Tyrosine Metabolism	0.069	0.155	0.329
Phospholipid Biosynthesis	0.069	0.325	0.506
Starch and Sucrose Metabolism	0.065	0.355	0.544
Pyruvate Metabolism	0.063	0.301	0.488
Citric Acid Cycle	0.063	0.370	0.549
Beta Oxidation of Very Long Chain Fatty Acids	0.059	0.504	0.676
Aspartate Metabolism	0.057	0.414	0.597
Valine, Leucine and Isoleucine Degradation	0.050	0.436	0.613
Porphyrin Metabolism	0.050	0.483	0.667
Catecholamine Biosynthesis	0.050	0.562	0.715
Ubiquinone Biosynthesis	0.050	0.562	0.715
Lactose Synthesis	0.050	0.562	0.715
Bile Acid Biosynthesis	0.046	0.490	0.667
Sulfate/Sulfite Metabolism	0.045	0.597	0.750
Arachidonic Acid Metabolism	0.043	0.532	0.704
Oxidation of Branched Chain Fatty Acids	0.038	0.659	0.807
Inositol Phosphate Metabolism	0.038	0.659	0.807
Pentose Phosphate Pathway	0.034	0.699	0.846
Inositol Metabolism	0.030	0.746	0.892
Steroid Biosynthesis	0.021	0.866	1.000
